# Decoding the Tumor-Associated Microbiota: From Origins to Nanomedicine Applications in Cancer Therapy

**DOI:** 10.3390/biology14030243

**Published:** 2025-02-27

**Authors:** Ruiqi Wang, Weizheng Li, Hongqian Cao, Lei Zhang

**Affiliations:** 1 Microbiome-X, School of Public Health, Cheeloo College of Medicine, Shandong University, Jinan 250012, China; 202216382@mail.sdu.edu.cn (R.W.); 202220988@mail.sdu.edu.cn (W.L.); 2 Department of Health Inspection and Quarantine, School of Public Health, Cheeloo College of Medicine, Shandong University, Jinan 250012, China; 3 State Key Laboratory of Microbial Technology, Shandong University, Qingdao 266237, China

**Keywords:** cancer therapy, tumor microenvironment, tumor-associated microbiota, nanotechnology, immune modulation, precision medicine

## Abstract

Growing evidence reveals that the tumor microbiome—comprising distinct microbial communities within neoplastic tissues—exerts a profound influence on cancer initiation, progression, and therapeutic response. These microbes actively reshape the tumor microenvironment (TME) through metabolite secretion, the modulation of immune pathways, and direct interactions with host cells, thereby affecting tumor biology and therapeutic outcomes. Despite substantial heterogeneity among cancer types, recent insights underscore the tumor microbiome’s potential as both a diagnostic/prognostic biomarker and a targetable component for innovative treatments. In this review, we synthesize emerging knowledge on the mechanistic roles of tumor-associated microbiota in shaping the TME, with a focus on how these discoveries can guide novel therapeutic strategies. We further explore interdisciplinary advances, including the convergence of microbiomics and nanotechnology, to enhance drug delivery, circumvent resistance, and foster TME remodeling. By highlighting these cutting-edge developments, our review underscores the transformative potential of integrating tumor microbiome research into precision oncology and advancing more personalized cancer therapies.

## Simple Summary

Recent discoveries reveal that tumors host unique communities of microbes, which significantly influence cancer growth, spread, and response to treatments. These microbes interact with cancer cells and their surroundings by releasing chemical substances, altering immune system activity, and directly affecting healthy tissues, thereby shaping how tumors behave and respond to therapy. We explore how these microbial communities differ between cancer types and can serve as indicators of disease progression and treatment success. This review highlights promising new therapeutic strategies that target these microbes, particularly through advances in nanotechnology that allow for the precise delivery of treatments to tumor sites. We also discuss key challenges in studying tumor microbes, including the need for standardized research methods and the careful validation of findings between laboratory models and human patients. This growing field of research offers exciting possibilities for developing more personalized cancer treatments by considering each patient’s unique tumor microbial profile, potentially transforming how we diagnose and treat cancer in the future.

## 1. Introduction

The microbiome encompasses bacteria, viruses, fungi, and other microbial entities, which are present in nearly all human organs and play indispensable roles in regulating host physiology and immune responses [1,2]. Advancements in sequencing technologies have enabled researchers to investigate the human microbiome with unprecedented depth, encompassing ecological niches traditionally deemed sterile, such as the lungs, pancreas, and mammary glands [3,4]. Recent studies employing high-sensitivity sequencing methods have identified low-abundance microbial communities within these sites, although subsequent sections address the challenges of contamination and bias in detecting low-biomass microorganisms. Notably, microbes have been detected in pathological regions, including tumors. Various tumor types, such as breast cancer, lung cancer, and pancreatic cancer [5], harbor these microorganisms. These microbial communities within tumors are referred to as the tumor microbiome, and their origins are highly complex [6]. The primary impact of microbes within tumors involves the modulation of the tumor microenvironment. These microorganisms can establish a unique metabolic and physiological milieu at the tumor site by secreting metabolic products, modulating immune responses, or interacting with host cells, thereby playing critical roles [7,8,9,10]. The relationship between tumor microbiota and tumor biology is multifaceted: microbes can both facilitate tumor initiation and progression under certain conditions and inhibit tumor growth through other mechanisms. Recent studies provide evidence that these microbes are not merely passive colonizers but actively influence cancer pathophysiology through various mechanisms. For example, the tumor microbiome can directly affect tumor initiation and progression by inducing chronic inflammation, promoting genomic instability, and modulating the immune microenvironment [11].

Microorganisms’ remarkable metabolic capabilities can significantly influence therapeutic processes. For example, tumor-associated microbiota can modulate the pharmacokinetics and pharmacodynamics of chemotherapeutic agents, thereby altering treatment efficacy and toxicity [12]. Furthermore, beyond metabolic interactions, microorganisms can modulate the host immune system, subtly affecting the overall efficacy of immunotherapies [13]. However, the biological significance and functional characteristics of tumor-associated microbial communities remain incompletely understood. Similarly to microbiomes in other ecological niches, intratumoral microbes can both promote cancer initiation and progression and potentially exert synergistic or antagonistic effects during treatment. In addition to exploring their roles in tumor pathogenesis, there is growing interest in leveraging tumor-associated microbiota for therapeutic interventions. Recent advancements in nanotechnology have enabled the precise delivery of microbe-derived or engineered nanoparticles to tumor tissues, demonstrating potential synergistic effects in remodeling the tumor microenvironment, enhancing drug delivery efficiency, and overcoming drug resistance [14]. The seamless integration of microbiomics and nanomedicine heralds a transformative era in cancer therapy, potentially leading to highly personalized and effective treatment strategies.

This review provides a comprehensive analysis of the complex interactions between tumor microbiota and the tumor microenvironment, emphasizing their combined roles in cancer progression and therapeutic responses. We synthesize current insights into the origins, functional contributions, and clinical implications of tumor-associated microbiota, focusing on their capacity to modulate tumorigenesis and therapy efficacy. Key discussions include advancements in microbial biomarker discovery for precision diagnostics and emerging nanotechnology-based approaches targeting tumor-associated microbes to enhance therapeutic efficacy [15]. By integrating these diverse aspects, this review underscores the pivotal role of tumor microbiota in shaping cancer dynamics and presents novel strategies that leverage microbial interactions to advance clinical interventions in oncology.

## 2. Tumor Microenvironment and Tumor Microbiota

TME is a dynamic and heterogeneous ecosystem that plays a pivotal role in tumor progression, invasion, and metastasis [16]. In addition to heterogeneous populations of malignant cells, the TME comprises stromal cells, infiltrating immune cells, vascular networks, and the extracellular matrix (ECM). These diverse components engage in intricate interactions that collectively regulate tumor progression [17]. Specifically, stromal cells and the ECM provide structural support and growth signals to tumor cells, while immune cell infiltration is critical in modulating tumor immune evasion. Vascular networks supply essential nutrients and oxygen to the tumor, facilitating the migratory and metastatic capabilities of cancer cells. Additionally, dynamic alterations within the TME, such as metabolic reprogramming and the establishment of an acidic milieu, promote angiogenesis and the recruitment of tumor-associated macrophages (TAMs) and fibroblasts, thereby accelerating tumor progression [16,18]. A hallmark of tumors is their ability to evade immune surveillance, primarily through the suppression of cytotoxic T cell activity and the regulation of macrophage functions, which fosters immune escape and sustained proliferation [19]. Notably, immune and stromal cells within the TME significantly contribute to tumor immune evasion and metabolic reprogramming, thereby influencing the tumor’s responsiveness to therapy [20]. Additionally, the TME communicates with tumor cells via exosomes, cytokines, and other molecular signals, enhancing tumor cell survival, immune resistance, and metastatic potential [21]. Immunosuppressive factors, including regulatory T cells (Tregs), myeloid-derived suppressor cells (MDSCs), and alternatively activated macrophages, are frequently prevalent within the TME, creating niches that support tumor survival, disrupt anti-tumor immunity, and promote immune tolerance [19,22].

In addition to cellular and molecular components, the diverse tumor-associated microbiota within the TME constitutes a crucial element, encompassing bacteria, fungi, and viruses [23,24]. These microbial communities are recognized as significant influencers of tumor biology and therapeutic responses. The presence of microorganisms within tumors is not a novel discovery; as early as the 19th century, hypotheses were proposed suggesting that microbes might play roles in cancer biology, thereby laying the foundation for subsequent investigations into intratumoral microbial communities and the development of microbiome-based immunotherapies [25]. A prominent example is *Helicobacter pylori*, regarded as one of the primary carcinogenic factors in gastric cancer. *H. pylori* contributes to gastric carcinogenesis by inducing chronic inflammation and altering cellular signaling pathways [26]. Furthermore, other microorganisms, such as *F. nucleatum*, are closely associated with the progression of colorectal cancer, promoting tumor development and malignant transformation by creating pro-inflammatory microenvironments and enhancing immune cell infiltration [27,28]. These examples illustrate how specific microbial species within the TME can influence distinct tumor types through diverse mechanisms. Advancements in next-generation sequencing and metagenomic technologies have significantly accelerated research on the tumor microbiome, enabling comprehensive analyses of microbial communities within tumors and enhancing our understanding of their diversity and functional roles. A pivotal study conducted in 2020 utilized next-generation sequencing alongside fluorescence in situ hybridization (FISH) to characterize the tumor microbiomes of 1526 tumor and adjacent normal samples across seven cancer types, including breast cancer, lung cancer, ovarian cancer, pancreatic cancer, melanoma, bone cancer, and brain cancer [5]. The findings revealed that each cancer type harbored a distinct microbial composition, with the breast tumor microbiome exhibiting particularly high diversity [5].

Emerging evidence suggests some similarities in tumor microbiome compositions between human patients and experimental animal models within specific tumor types [29,30]. However, the frequent enrichment of food-associated bacteria, particularly *Agrobacterium* and *Rhizobium* genera [31], in animal tumor samples highlights the potential influence of host dietary patterns, specifically the distinct differences between human omnivorous diets and standardized laboratory animal feed, in shaping tumor-associated microbial communities.

Furthermore, the characterization of microbial profiles exhibits methodological dependencies across various detection platforms. While techniques such as 16S rRNA sequencing, metagenomic sequencing, and FISH each offer distinct advantages in detection sensitivity, taxonomic resolution, and spatial localization capabilities, they yield partially overlapping yet non-identical microbial signatures across studies [32,33]. This technical heterogeneity underscores a fundamental challenge in tumor microbiome research: the critical need for standardized approaches in microbiome sequencing and functional characterization methodologies. The microbial composition of different cancers is shown in Table 1.

Age represents a critical determinant in tumor biology, fundamentally influencing both tumor development and progression. Recent investigations have revealed that age gradients substantially modulate both the compositional dynamics and metabolic architecture of the tumor microbiome. In a pivotal study of oral squamous cell carcinoma (OSCC), Zhang et al. [63] demonstrated distinct age-dependent microbial signatures: younger patients (<50 years) exhibited significant enrichment of *Ralstonia*, *Prevotella*, and *Ochrobactrum* genera, while elderly patients (>60 years) showed predominant *Pedobacter* colonization, correlating with age-specific metabolic pathway alterations.

This age-associated microbial restructuring extends to colorectal cancer models, where aged ApcMin/+; III0−/− mice demonstrated a significantly enhanced colon tumor burden compared to younger counterparts, mechanistically linked to the enrichment of colibactin-producing *Escherichia coli* (pks+ *E. coli*) within the intestinal microbiota [64]. These age-dependent microbiome alterations establish a self-perpetuating cycle, simultaneously driving tumorigenesis while promoting systemic inflammation and immunosenescence.

The progression of aging correlates with diminishing intestinal microbial diversity and the accelerated depletion of beneficial bacterial populations, compromising mucosal barrier integrity and perpetuating chronic inflammation. Biragyn and Ferrucci [65] documented significant reductions in short-chain fatty acid (SCFA)-producing bacteria, notably *Akkermansia muciniphila* and *Faecalibacterium prausnitzii*, in elderly populations, concurrent with an increased abundance of pro-inflammatory genera such as *Alistipes* and *Enterobacteriaceae*. Mechanistic studies in aged mouse models revealed that SCFA depletion compromises hypoxia-inducible factor (HIF) stability in intestinal epithelial cells, deteriorating barrier function and facilitating lipopolysaccharide (LPS) translocation and subsequent TLR4-mediated inflammatory cascade activation [66]. This “inflammaging” phenotype establishes a pro-tumorigenic microenvironment through the sustained production of inflammatory mediators, including IL-6 and TNF-α, culminating in DNA damage accumulation and apoptotic resistance [65].

## 3. Profiling Tumor Microbiota: Techniques and Challenges

Building upon initial investigations into the sources and colonization pathways of microorganisms within the TME, the precise identification and characterization of these microbial communities’ composition and function are crucial for a comprehensive understanding of tumor biology. However, accurately profiling the microbiota within tumors remains a significant challenge due to the inherent complexity and heterogeneity of tumor tissues, low microbial biomass, high background noise from host DNA, and susceptibility to contamination during sample processing.

**Figure 1:**
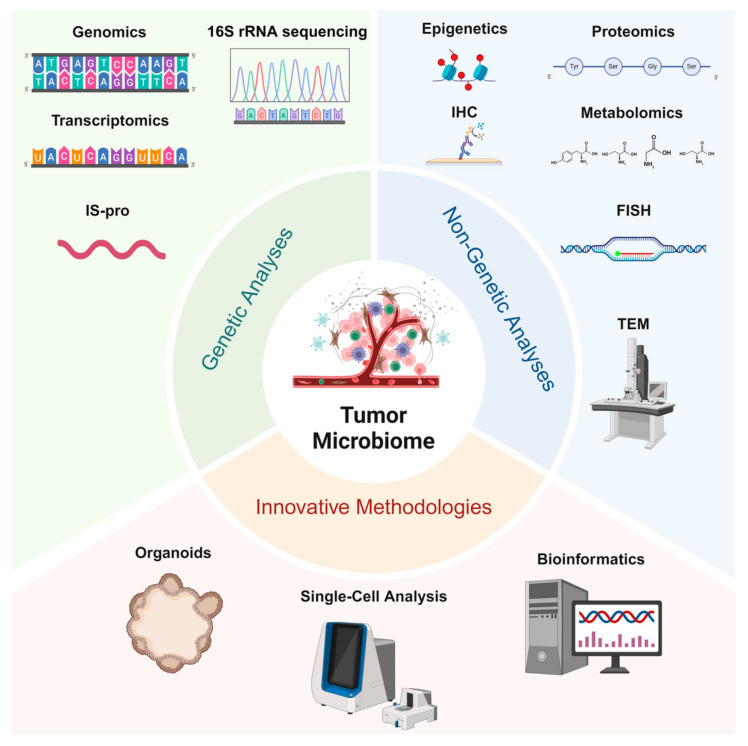
Studying tumor microbiomes involves genetic analyses (genomics, 16S rRNA sequencing, transcriptomics, and IS-pro profiling) for microbial identification and functional insights, non-genetic analyses (proteomics, metabolomics, IHC, FISH, and TEM) to examine microbial–tumor interactions, and innovative methodologies (organoids, single-cell analysis, and bioinformatics) that enable the advanced exploration of tumor–microbe dynamics and support translational research.

In recent years, significant advancements in the identification of tumor-associated microbiomes have been driven not only by the development of traditional genetic and non-genetic analytical methods but also by the emergence of innovative technologies such as single-cell analysis and organoid models, which provide robust platforms for in-depth investigation (Figure 1).

### 3.1. Genetic Analyses

High-throughput 16S rRNA gene sequencing provides rapid taxonomic composition information at a relatively low cost [67,68]. However, its reliance on existing reference databases and limited resolution at the species or strain level highlight the need for complementary approaches. Metagenomic sequencing addresses these limitations by enabling the comprehensive analysis of genomic features, facilitating the discovery of previously unknown species and potential functional genes [69]. Huang et al. employed metagenomic analysis to elucidate both the classification and functional roles of the microbiota, as well as to assess the response to neoadjuvant chemoradiotherapy in patients with advanced colorectal cancer [70]. Metatranscriptomics delves into the active metabolic pathways and gene expression profiles of microbes within tumors [71], offering critical insights into their functional roles. Additionally, the Intergenic Spacer Profiling (IS-pro) technique targets intergenic spacer regions, delivering a rapid and high-resolution method for community analysis that complements traditional molecular marker approaches [72]. Together, these genomic tools form a robust platform for decoding the microbial diversity and functional dynamics in tumor microenvironments.

### 3.2. Non-Genetic Analyses

Beyond genetic methodologies, proteomics and metabolomics precisely delineate the molecular interactions between microorganisms and tumor cells, revealing potential metabolic couplings and protein regulatory mechanisms [73]. Methylglyoxal (MGO) is a microbial metabolite enzymatically synthesized by MGS [74], and Devin M. Ray et al. elucidated the regulatory role of MGO in the DNA-templating process through the application of quantitative proteomics [75]. Furthermore, epigenomic approaches examine epigenetic alterations, including DNA methylation, histone modifications, and non-coding RNA regulation, providing new perspectives on the synergistic effects of host–microbe interactions in tumor progression [76]. Immunohistochemistry (IHC) employs the specific binding of antigens and antibodies, utilizing enzymes, e.g., Horseradish Peroxidase(HRP) or fluorescent molecules conjugated to antibodies for colorimetric or fluorescent labeling, thereby enabling the visual detection of specific protein expression and localization in tissue sections [77]. Similarly, fluorescence in situ hybridization FISH uses fluorescence-labeled nucleic acid probes that hybridize with the chromosomes or DNA sequences of tumor cells, allowing the observation of genetic or chromosomal abnormalities—such as amplifications, deletions, or fusions—under a fluorescence microscope [78]. In a pivotal study conducted in 2020, FISH technology was employed to analyze 1526 tumors spanning seven distinct cancer types, along with their adjacent normal tissues [5]. Additionally, correlative light and electron microscopy (CLEM) and transmission electron microscopy (TEM) offer high-resolution imaging capabilities to examine the ultrastructural and intimate associations between microorganisms and tumor cells [79,80]. In 2022, using high-resolution electron microscopy, Cai Shang and colleagues provided direct visual evidence of bacterial localization within the cytoplasm of tumor cells in a spontaneous mouse breast cancer model, thereby illuminating previously uncharacterized structural features of tumor-associated microbes [29]. Collectively, these techniques complement genomic methods by offering spatial and molecular perspectives crucial for understanding microbe–tumor interactions.

### 3.3. Innovative Methodologies

Emerging technologies such as single-cell analysis and patient-derived organoids (PDOs) offer promising solutions. Single-cell analytical techniques, which integrate genomic, transcriptomic, and proteomic data [80], offer a more nuanced understanding of the heterogeneity and functional dynamics of tumor-associated microbiota. Concurrently, the synergistic integration of multi-omics data facilitates the construction of comprehensive tumor–microbe interaction maps, laying the foundation for elucidating the complex biological networks that drive tumor progression [73]. Driven by robust bioinformatics tools and interdisciplinary collaboration, the continuous refinement of these analytical strategies will enhance our understanding of the potential impacts of tumor microbiota on cancer diagnosis, prognosis, and therapy. For instance, Bassel Ghaddar et al. utilized single-cell transcriptome and microbiome correlation analysis to explore host–microbe interactions in pancreatic cancer [81]. Additionally, PDOs have significantly advanced tumor microbiome research by providing a three-dimensional culture system that closely mimics the in vivo tumor environment [82,83]. Carmen Aguilar et al. discovered that *H. pylori* preferentially targets highly differentiated pit cells in human stem cell-derived gastric organoids [84]. These organoid models not only elucidate the complex interactions between tumors and microorganisms but also offer insights into the role of microorganisms in cancer pathogenesis, drug resistance, and treatment response, thereby providing a robust platform for developing personalized medical strategies.

### 3.4. Challenges and Limitations

Despite significant advancements, each analytical technique presents specific limitations. For example, 16S rRNA sequencing is dependent on existing reference databases and may be subject to amplification biases [85,86]. Metagenomic analyses, on the other hand, require substantial computational resources [87,88]. IS-pro technology demands specialized operator expertise [72]. Additionally, various mass spectrometers used in proteomic analyses differ in sensitivity, resolution, and accuracy for detecting differential protein expression. For instance, instruments with lower resolution may struggle to distinguish peptides with similar masses, thereby reducing identification accuracy [89,90]. Protein samples typically require enzymatic digestion, and certain proteases are sensitive to reaction conditions such as temperature, pH, and ionic strength. Even minor deviations can result in decreased enzymatic efficiency or non-specific cleavage, impacting the accuracy of subsequent analyses [91]. Metabolomics faces challenges in dynamic observation due to the difficulty in capturing transient metabolites [92,93].

However, analyzing tumor-associated microbiota demands rigorous protocols and meticulous data processing to ensure credible conclusions. A pivotal study published in 2020 (now retracted), drawing on TCGA datasets, proposed that certain cancer types exhibit distinctive microbiome signatures, which can also be detected in blood [94]. Although these findings sparked enthusiasm for the potential of noninvasive cancer diagnoses, the work was subjected to intense scrutiny and was subsequently retracted. Gihawi et al. questioned whether human DNA contamination in reference databases could lead to the misclassification of microbial signals and whether batch correction methods might introduce artificial signals, thereby inflating machine-learning performance [95]. These controversies underscore the complexity and potential pitfalls of dealing with low-abundance microbial data, highlighting the importance of caution when interpreting results. Subsequent clarifications, incorporating more stringent database cleanup tools (such as Exhaustive) and comprehensive host-filtering strategies, revealed that the observed cancer type-specific microbial signals remained consistent under different conditions [96]. Furthermore, even without batch correction, the classifier’s performance did not markedly decline, supporting the core conclusions of the original publication [96]. Nonetheless, the fundamental methodological concerns leading to the retraction of the original study emphasize critical considerations for the field. This debate has illuminated possible paths for improving data analysis methods in tumor microbiome research. It emphasizes that database assembly, contamination checks, and robust normalization across multi-institutional datasets are not mere technicalities but rather indispensable elements for drawing reliable scientific inferences. Recognizing these factors, the tumor microbiome field urgently requires standardized protocols, and this underscores the necessity of stringent data quality control, particularly for low-abundance signals.

Looking ahead, researchers should foster advancements in database cleaning, batch correction, and refined signal detection tools, integrating innovations from biology, statistics, and artificial intelligence to enhance analytical precision. Beyond molecular and computational techniques, cutting-edge imaging modalities ought to be incorporated into tumor microbiome investigations to strengthen the reliability and interpretability of findings. Methods such as confocal laser scanning microscopy (CLSM), electron microscopy (EM), and correlative light and CLEM can provide spatial resolution and high-definition visualization of microbial communities within tumor tissues. Directly observing microbial localization, distribution, and interactions with host cells offers a valuable complement to molecular analyses. For example, combining FISH with CLSM allows for the precise detection of microbial species in a heterogeneous tumor microenvironment, whereas CLEM bridges the gap between molecular specificity and ultrastructural context. Nevertheless, these approaches are not without limitations: IHC and FISH necessitate high-quality tissue samples and the availability of specific probes or antibodies [97], preparing samples for high-resolution imaging tends to be time-consuming and susceptible to artifacts, and interpreting intricate images calls for considerable expertise. Consequently, integrating genomic analysis, imaging data, and multi-omics approaches provides a more holistic and substantiated perspective on interactions between microbes and tumors. This multimodal strategy is critical for enhancing the robustness of conclusions and minimizing the biases associated with any single technique. Although the promise of tumor-specific microbial signatures is substantial, acknowledging the field’s technical constraints and employing painstaking research practices remain paramount. In the end, progress in tumor microbiome studies depends on sustained interdisciplinary collaboration, rigorous experimental design, and the continual refinement of data processing pipelines, all of which will elevate the reproducibility and reliability of future outcomes.

## 4. Origins and Functional Roles of Tumor Microbiota

**Figure 2:**
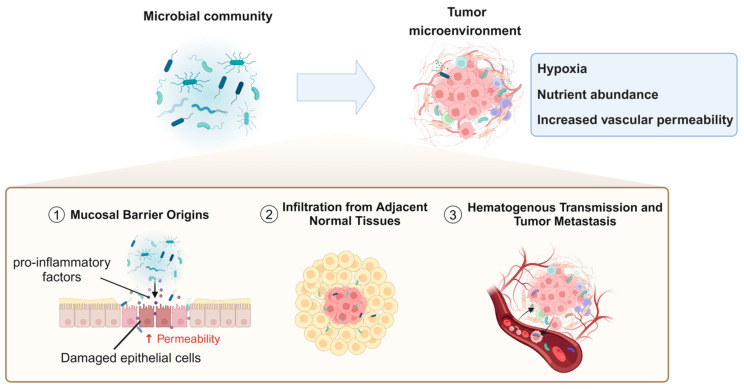
Mechanistic pathways of microbial infiltration and colonization in the tumor microenvironment.

In recent years, numerous studies have identified a variety of bacterial species within the TME. However, the origins, colonization processes, and specific mechanistic roles of these bacteria in tumor biology remain incompletely understood [98,99]. Currently, the potential sources of tumor-associated bacteria primarily include the following pathways: mucosal barrier origins, infiltration from adjacent normal tissues, hematogenous transmission, and tumor metastasis (Figure 2).

### 4.1. Mucosal Barrier Origins

Commensal and pathogenic microorganisms typically reside at mucosal barrier interfaces, where these barriers function to protect the body by preventing the entry of microbes or harmful substances into deeper tissues. However, under inflammatory conditions or other pathological states that compromise the integrity of these barriers, bacteria can more easily migrate through damaged epithelial cell junctions or permeable vascular walls into underlying tissues, including tumors [100,101]. Additionally, certain pathogens may directly induce inflammation or disrupt mucosal barriers. For instance, the destruction of the gastrointestinal barrier is closely associated with the presence of *B. fragilis* in colorectal tumors. *B. fragilis* can provoke inflammatory responses that facilitate carcinogenesis [102]. Tjalsma et al. [27] proposed the “bacterial driver–passenger model”, which hypothesizes that intestinal bacteria play sequential roles in the initiation and progression of colorectal cancer (CRC). Initially, “driver” bacteria, such as *enterotoxigenic Bacteroides fragilis* (ETBF) and specific *E. coli* strains, initiate tumorigenesis by producing genotoxins, inducing inflammation, and causing DNA damage, leading to the formation of malignant adenomas. As CRC progresses, the TME undergoes changes that favor the growth of “passenger” bacteria, such as *F. nucleatum* and *Streptococcus gallolyticus subsp. gallolyticus*. These “passenger” bacteria adapt to the altered niche and may further influence tumor progression through pro-inflammatory or other tumor-modulating mechanisms. Due to their tumor–microbe properties, they have the potential to become noninvasive diagnostic tools. In addition, from a therapeutic perspective, probiotics or engineered microbial therapies can restore mucosal integrity, reduce inflammation, and mitigate tumor-promoting effects.

### 4.2. Infiltration from Adjacent Normal Tissues

Beyond migration through compromised epithelial barriers or permeable vasculature, adjacent normal tissues may serve as reservoirs for tumor-associated microbiota, allowing bacteria to infiltrate tumor sites through diffusion. Studies have provided evidence that the microbial composition of normal adjacent tissues (NATs) closely resembles that of malignant tissues [5].

### 4.3. Hematogenous Transmission and Tumor Metastasis

Hematogenous transmission represents another significant pathway, wherein bacteria can colonize tumors by exploiting the increased vascular permeability and retention effects commonly observed in tumor vasculature [103]. For example, the tumor-resident bacterium *E. coli* has been shown to disrupt the intestinal vascular barrier, promoting its dissemination to distal organs such as the liver and facilitating the formation of pre-metastatic niches (PMNs) [104].

During the metastatic process, bacteria may be co-transferred with cancer cells to distant sites, introducing microbes into secondary tumor locations. This further supports the potential for microbial dissemination via the bloodstream, as studies have demonstrated a high consistency of Fusobacterium and its associated microbiota in primary colorectal tumors and their liver metastases. Moreover, research indicates that Fusobacterium may be introduced into metastatic lesions alongside migrating cancer cells, thereby promoting tumor growth and dissemination [105].

Bacteria may reorganize the actin cytoskeleton of cancer cells, enhancing their resistance to fluid shear stress, thereby increasing their survival in the circulatory system and promoting metastasis [29]. Aikun Fu et al. have used spontaneous mouse models of breast cancer (MMTV-PyMT) to explore the role of tumor-resident bacteria in metastasis. These studies found that the elimination of intratumoral bacteria significantly reduced lung metastasis without markedly affecting the growth of primary tumors, suggesting that bacteria play a crucial role in the metastatic process [29]. Additionally, the ecological environment of tumors must be considered, as bacterial colonization within tumors is likely influenced by intrinsic factors of the TME, such as hypoxia, increased vascular permeability, and nutrient abundance [106,107,108]. Furthermore, the TME can provide an environment that protects bacteria from immune clearance, collectively creating favorable conditions for microbial survival and proliferation.

The origins of the tumor microbiome are influenced by multiple factors. Current research emphasizes the complexity of microbe–tumor interactions, making it crucial to understand the sources of microorganisms within tumors. This understanding is vital for elucidating the causal relationships between microbial infiltration and colonization in the process of tumorigenesis. The microbes detected within tumor tissues may not be merely random “passengers”, but rather integral components closely associated with tumor physiology, local immune status, and metabolic activities. Identifying the sources of these microbes aids in preliminarily determining their roles in tumor formation and progression. Furthermore, distinguishing the origins of microbes is essential for differentiating between “opportunistic invaders” and “endogenous partners”. If certain microbes originate from adjacent normal tissues or mucosal microbiota, this may indicate a disruption in the succession and balance between normal and abnormal states. Under specific conditions, these microbes may thrive and overproliferate, with their metabolic products, virulence factors, and interactions with the immune system potentially promoting tumor cell growth and metastasis. Conversely, if some microbes originate from external environments (such as the oral cavity, skin, or through external infections), it suggests that researchers should focus on the association between environmental factors and tumorigenesis, thereby guiding clinical prevention and intervention strategies. By thoroughly understanding how microbes enter and colonize tumor tissues, we can reevaluate the comprehensive landscape of tumor development. This insight not only facilitates a more precise understanding of the complex network structure of the tumor microenvironment but also provides a reliable theoretical foundation and potential intervention targets for future precision diagnostics and therapeutics.

## 5. The Role of the Microbiome in Cancer

**Figure 3:**
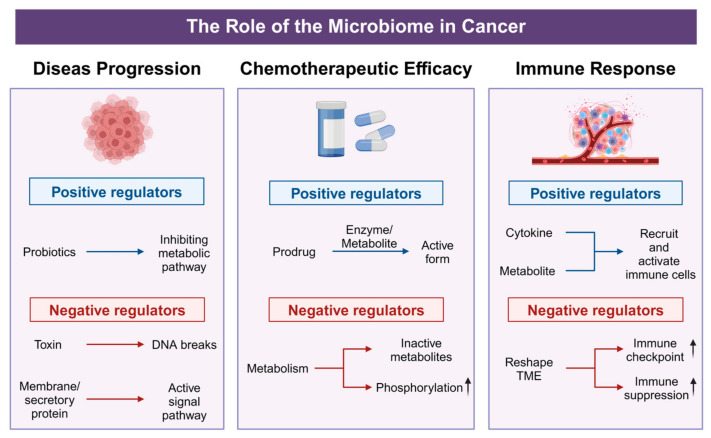
Positive and negative effects of microorganisms on tumor progression. In disease progression, probiotics act as positive regulators by suppressing tumor-associated metabolic pathways, whereas bacterial toxins and membrane/secretory proteins function as negative regulators, inducing DNA break or activating signal pathway. For chemotherapeutic efficacy, beneficial metabolites enhance drug activity through prodrug activation, while harmful microbes reduce therapeutic potency by promoting phosphorylation-mediated drug inactivation or converting agents into inactive metabolites. In immune regulation, beneficial microbes bolster anti-tumor immunity via cytokine-driven recruitment and activation of immune cells, whereas pathogenic bacteria upregulate immune checkpoints and drive immune suppression.

The intricate interplay between tumor-associated microbiota and host physiology has emerged as a pivotal factor in cancer biology and treatment. Recent investigations reveal that these microbial communities not only contribute to tumor initiation and progression but also significantly modulate chemotherapeutic efficacy and immune responses. By secreting metabolites, reshaping cytokine and chemokine profiles, and affecting immune cell infiltration, the tumor microbiome can enhance or diminish the efficacy of conventional and targeted therapies (Figure 3). These emerging insights underscore the microbiome’s dual potential as both a biomarker for disease prognosis and a modifiable target for therapeutic interventions. The following sections offer a detailed exploration of how tumor-associated microbial populations influence cancer progression, alter drug metabolism, and modulate the tumor immune microenvironment—shedding light on new avenues for precision oncology and personalized treatment strategies.

### 5.1. Impact on Disease Progression

The tumor microbiome plays multifaceted and critical roles in cancer progression, exhibiting both tumor-promoting and tumor-suppressing activities that significantly vary across different cancer types and microbial communities. Specifically, certain microorganisms facilitate tumorigenesis and tumor progression through mechanisms such as inducing DNA damage [109], activating cancer-associated signaling pathways [110], regulating cytokine production [111], and altering drug metabolism [112]. For instance, *E. coli* strains that produce the genotoxin colibactin have been shown to cause DNA double-strand breaks, thereby exacerbating genetic mutations and directly promoting tumor formation [113]. Similarly, *F. nucleatum* can activate the Wnt/β-catenin and NF-κB signaling pathways, leading to increased cellular proliferation and inhibited apoptosis, collectively fostering tumor growth [35]. Lactic acid bacteria inhibit the progression of colorectal cancer by downregulating the metabolism of glycine, serine, and threonine to reduce sphingomyelin [114]. In summary, these diverse interactions underscore the tumor microbiome’s multifaceted influence on cancer progression, highlighting its potential as a target for therapeutic strategies and as a biomarker for disease prognosis.

### 5.2. Influence on Chemotherapeutic Efficacy

Building on previous research within the gut microbiome, the metabolic diversity of microorganisms has demonstrated significant potential in modulating the efficacy of cancer therapies. These microorganisms can either enhance or impair the effectiveness of chemotherapeutic agents (such as irinotecan, oxaliplatin, cyclophosphamide, 5-fluorouracil, gemcitabine, and anthracyclines), immunotherapies (such as anti-PD1 and anti-CTLA4 antibodies), and radiotherapy [115]. Under the metabolic influence of *E. coli* and certain gut microbiota, bile acids can be converted into deoxycholic acid (DCA) [116]. DCA upregulates pro-inflammatory cytokines and immune regulatory markers and promotes STAT3 phosphorylation, nuclear accumulation, and the binding of STAT3 to the KLF5 promoter [117], thereby creating conditions conducive to carcinogenesis. This microbial metabolic diversity further elucidates how the complex and dynamic TME influences cancer treatment responses through multiple mechanisms. The TME encompasses not only cellular components, soluble factors, and ECM dynamics but also the coordinated mechanisms of immune evasion and therapeutic resistance [118]. As a crucial component of the TME, the intratumoral microbiota plays a key role in its formation and maintenance, significantly impacting the efficacy of anti-tumor therapies through intricate metabolic networks and immune regulatory mechanisms.

Studies have demonstrated that the tumor microbiome in pancreatic ductal adenocarcinoma, such as *Gammaproteobacteria*, can metabolize chemotherapeutic agents like gemcitabine (2′,2′-difluorodeoxycytidine) into inactive forms, thereby reducing their efficacy [119]. Similarly, certain gut bacteria, including those from the Bacteroides genus, can metabolize chemotherapeutic drugs such as irinotecan into inactive metabolites, thereby diminishing their therapeutic effects [120]. Additionally, research has found that tumor-resident *Lactobacillus* species in cervical cancer patients induce metabolic reprogramming through lactate production, enhancing glycolysis, oxidative stress pathways, and hypoxia signaling within the TME. This results in elevated lactate levels, promoting resistance to chemotherapy and radiotherapy [121]. Similarly, analogous phenomena have been observed in fungi; studies indicate that fungal migration from the gut to the pancreas facilitates the progression of pancreatic ductal adenocarcinoma, while clearance of these fungi (e.g., using amphotericin B) enhances the efficacy of gemcitabine chemotherapy and reduces drug resistance [122]. The metabolic activities of these microorganisms significantly increase resistance responses to anti-cancer drugs, particularly when bacterial populations evolve mechanisms to evade or alter drug actions, ultimately leading to diminished therapeutic outcomes. Notably, these adverse effects can be modulated by combining antibiotics with anti-cancer therapies, as the targeted alteration of the microbiota composition may help restore drug sensitivity and improve overall treatment efficacy [119,123,124]. However, not all microorganisms inhibit chemotherapy. For instance, *Clostridium novyi* can convert prodrug forms of chemotherapeutic agents, such as 5-fluorouracil (5-FU), into their active, anti-cancer forms, thereby enhancing the drug’s toxicity at tumor sites [120]. *Bifidobacterium* can enhance the anti-tumor effect of targeted drugs against the PD-1 and CTLA-4 molecules [125]. Additionally, microbial communities can influence the epithelial-to-mesenchymal transition (EMT) process. *B. fragilis*, for example, promotes the expression of Snail and Slug while suppressing the expression of the cell adhesion molecule E-cadherin, thereby increasing the metastatic potential of cancer cells [126].

### 5.3. Effects on Immune Response

Beyond their impact on chemotherapeutic agents, the intricate relationship between tumor-associated microbiota and cancer therapy is recognized as a key factor influencing the efficacy of immunotherapies. Tumor-associated microbiota can significantly regulate both innate and adaptive immune responses; specific microbial signatures can attract or repel immune cell infiltration, alter antigen presentation, and modulate cytokine and chemokine profiles within the tumor environment. Consequently, intratumoral bacteria establish an immunosuppressive niche that not only hinders conventional treatments such as chemotherapy and radiotherapy but also undermines the efficacy of immune checkpoint inhibitors (ICIs) and adoptive T cell therapies. *F. nucleatum* has been reported to impair immune surveillance and weaken anti-tumor immune responses by reducing T cell infiltration and activating pro-inflammatory pathways such as TLR4 signaling, thereby promoting tumor growth, immune evasion, and metastasis in colorectal cancer [105]. Additionally, *B. fragilis* can induce the production of interleukin-17 (IL-17), which fosters tumor growth through a chronic inflammatory environment [127]. Microbiome-induced immune modulation plays a crucial role in determining the efficacy of ICIs and other immunotherapeutic strategies. For example, Marlies Meisel et al. increased CD8+ Tc1 cells, inhibited tumor growth, and prolonged mouse survival by the intratumoral injection of *Lactobacillus reuteri* [128]. The presence of *Bifidobacterium* in the gut microbiome has been demonstrated to enhance CD8^+^ T cell infiltration and activation, thereby augmenting the efficacy of the PD-L1 checkpoint blockade in melanoma mouse models [129]. Similarly, specific microbial taxa such as *A. muciniphila* have been associated with improved responses to ICIs [130]. Conversely, certain microbial species can enhance anti-tumor immunity by activating cytotoxic T cells and natural killer (NK) cells, thereby inhibiting tumor progression. Examples include *Bifidobacterium* [125,131] and *A. muciniphila* [131]. *Lactobacillus* stimulates the production of anti-inflammatory cytokines, supporting anti-tumor immunity and inhibiting cancer progression [132]. Moreover, short-chain fatty acids (SCFAs) such as butyrate, produced by *Roseburia intestinalis*, have been shown to inhibit histone deacetylases [133,134], thereby altering gene expression and suppressing tumor growth. These studies demonstrate that tumor-associated microbiotas regulate immune responses through multiple mechanisms, influencing the efficacy of immunotherapies. The targeted modulation of microbiota composition holds promise for optimizing immunotherapeutic strategies and enhancing treatment outcomes.

## 6. Microbial Biomarkers for Precision Oncology Diagnostics

Tumor-associated microbiotas have emerged as a promising focus in precision oncology, offering insights into tumorigenesis, progression, and therapeutic responses [24]. Distinct microbial community profiles are frequently associated with specific tumor types and subtypes, highlighting their diagnostic potential [135]. These profiles are not only implicated in disease initiation and progression but also closely linked to patient prognosis [135,136,137]. In contrast to the relatively stable and balanced microbial ecology observed in healthy tissues, the microbial composition within tumor tissues often undergoes significant alterations, allowing specific microbial signatures to function as potential diagnostic biomarkers [138,139]. For example, research has shown that identifying specific bacterial marker groups within lung cancer tissues can differentiate patients with recurrence or metastasis from those without recurrence or metastasis. Bacterial genera such as *Acidovorax*, *Clostridioides*, *Succinimonas*, *Shewanella*, *Leuconostoc*, and *Dickeya* are closely associated with tumor staging, survival duration, and disease severity [140]. Additionally, a study on pancreatic ductal adenocarcinoma (PDAC) revealed that tumor tissues from long-term survivors (LTS) exhibited greater microbial diversity compared to short-term survivors (STS). Specific strains, including *Pseudoxanthomonas*, *Streptomyces*, *Saccharopolyspora*, and *Bacillus clausii*, demonstrated robust prognostic capabilities across multiple independent cohorts [59]. Furthermore, the features of the gut and respiratory microbiomes can offer valuable insights into the efficacy of ICIs. In patients with non-small-cell lung cancer (NSCLC), higher α-diversity within the gut microbiome is associated with improved responses to ICI therapy and extended progression-free survival. Conversely, patients who respond favorably to ICIs are more likely to harbor beneficial bacterial strains such as *Alistipes*, *Faecalibacterium*, and *Bifidobacterium*, whereas those with poor responses are enriched with potentially detrimental bacteria like *Fusobacterium* [141].

These findings highlight the potential of tumor-associated microbiota as diagnostic biomarkers and predictors of therapeutic outcomes, paving the way for more personalized and effective cancer management strategies. Moreover, fecal microbiota transplantation (FMT) using donors from LTSs has been shown in preclinical models to enhance the immune microenvironment and inhibit tumor growth, indicating that tumor-associated microbiota are not merely passive markers but may play a causal role in tumor progression [59]. In serous ovarian cystadenocarcinoma, microbial markers such as *Simiduia*, *Halolamina*, *Brachymonas*, and *Terasakiella* are associated with better survival prognosis, whereas markers like *Mitsuokella*, *Luteimonas*, *Magnetospirillum*, *Erwinia*, and *Salinisphaera* are closely linked to poorer outcomes. These findings provide valuable references for utilizing microbial patterns to predict clinical outcomes [142]. Intriguingly, the relevance of microbial biomarkers extends beyond bacterial communities, with fungal communities garnering increasing attention. For instance, *Aspergillus sydowii*-colonizing tumor tissues can exacerbate lung adenocarcinoma (LUAD) by modulating the TME, offering new perspectives for incorporating fungal biomarkers into diagnostic and therapeutic contexts [143]. However, translating these microbial features into reliable diagnostic tools remains challenging. Low-biomass and high host background DNA signals can result in contamination and false positives during sequencing and analysis processes [144,145,146]. Moreover, the dynamic nature of microbial communities within the tumor TME may respond to ongoing treatments and local environmental changes, complicating the establishment of standardized and reproducible detection protocols [147,148,149]. To address these challenges, future efforts must rigorously control sampling and processing workflows, improve sequencing and quantification methods, and integrate multi-omics data—including genomics, transcriptomics, metabolomics, and proteomics—to develop a more robust molecular framework for identifying and interpreting microbial characteristics [150,151]. Multidisciplinary collaboration and prospective clinical trial validations are essential for enhancing the predictive accuracy and practicality of microbial biomarkers [152,153,154]. Furthermore, prospective clinical trials are essential to validate microbial signatures in diverse patient populations and assess their predictive accuracy for therapeutic responses. Ultimately, these advancements aim to incorporate microbial signatures into precision oncology diagnostic and classification systems, thereby optimizing personalized treatment strategies and patient prognosis management.

## 7. Nanotechnology-Enhanced Microbial Therapies for Precision Oncology

In recent years, tumor microbiota research has increasingly transitioned from mere observation and identification to active regulation and practical application [6]. This shift not only underscores the growing synergy between microbiomics and oncology but also opens new avenues for developing innovative therapeutic strategies. Harnessing rapid advancements in nanotechnology, scientists have begun engineering microorganisms or their derivatives, enhancing these biological carriers with novel functionalities for tumor diagnosis and treatment [155].

**Figure 4:**
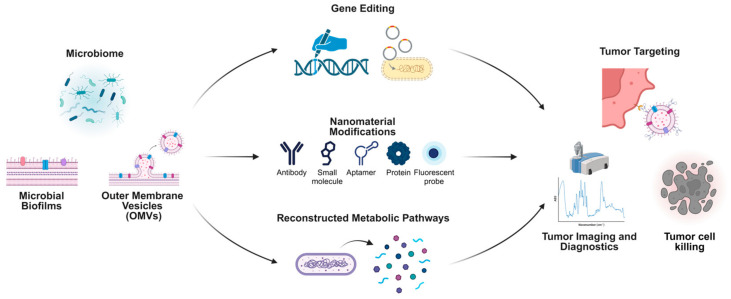
Microbiome-derived approaches integrated with nanotechnology for tumor-targeting and therapeutic applications.

There are comprehensive strategies leveraging microbiome-based systems and nanotechnology for cancer diagnosis and treatment. Microbial biofilms, OMVs, and direct microbiome modifications serve as foundational elements to engineer innovative therapeutic platforms. Gene-editing technologies enable the precise modification of microbial genomes, enhancing their therapeutic potential through the creation of designer microbes. Nanomaterial modifications, such as antibodies, small molecules, aptamers, proteins, and fluorescent probes, expand the functionality of microbiome-derived components for tumor targeting and imaging. Reconstructed metabolic pathways derived from microbial systems further enhance therapeutic payload delivery and specificity. These advancements collectively facilitate targeted tumor imaging, diagnostics, and the development of highly effective tumor-killing strategies, exemplifying the intersection of microbiome science and nanotechnology for cutting-edge oncology research (Figure 4).

Through genetic editing [156,157], metabolic pathway recombination, or the surface molecule modification [158,159] of specific bacterial strains, researchers can achieve highly selective colonization within tumor tissues. These engineered microorganisms are designed to express or release proteins, metabolites, and signaling molecules in a controlled manner. They can carry reporter genes or generate detectable signals within tumors, thereby improving the accuracy of in situ imaging and localization [160,161,162,163]. Moreover, these strains can undergo intelligent lysis in response to external stimuli, releasing anti-tumor drugs or immunomodulatory molecules directly into the tumor microenvironment. This strategy enables a targeted, efficient, and dynamically controllable drug delivery system [164,165,166]. The distinct advantage of microbial therapeutic approaches lies in their capacity to overcome the limitations associated with traditional drug delivery methods regarding dosage, timing, and precision, while fully leveraging the inherent biological properties of microorganisms to enhance the accuracy and safety of the treatment process.

Concurrently, nanomaterial platforms derived from microbiome components, such as bacterial outer membrane vesicles (OMVs), biofilms, and related molecules, are forging new pathways for tumor therapy and diagnosis. By extracting, purifying, and engineering OMVs [167], bacterial biofilms [168,169], and their specific components [170], researchers can optimize these nanostructures for more targeted accumulation within tumor tissues. These nanocomponents are pivotal in delivering anti-cancer drugs, imaging probes, and immunomodulatory agents. Typically, these nanoscale constructs exhibit excellent biocompatibility and physicochemical stability. Through precise modifications of their surface proteins and polysaccharide structures, they can specifically interact with tumor cells or components of the tumor extracellular matrix [167,171]. Studies have demonstrated that loading OMVs from tumor-homing bacterial strains with anti-cancer drugs or photosensitizers significantly enhances drug accumulation within tumors by exploiting the enhanced vascular permeability of tumor tissues and the inherent homing properties of bacteria. This strategy achieves more precise therapeutic effects while minimizing systemic toxic side effects [172,173]. Moreover, microbiome-derived nanoplatforms have shown substantial potential in imaging diagnostics. For instance, attaching fluorescent labels or magnetic nanoparticles to the surface of OMVs enables the high-sensitivity monitoring of tumor location, size, and growth dynamics in vivo [174,175]. These technologies can be integrated with existing imaging modalities, such as Magnetic Resonance Imaging (MRI) and fluorescence imaging, to provide multidimensional information for the early and accurate diagnosis of tumors [163,174,176]. Additionally, microbial-derived carriers can modulate the tumor immune microenvironment, further enhancing therapeutic outcomes. Research indicates that OMVs can alter the proportion and activity of regulatory T cells at tumor sites, induce immune cell subsets with greater anti-tumor potential, and enhance in vivo anti-cancer immune responses [177,178]. As our understanding of the structural and functional aspects of the tumor microbiome deepens, and as tools in synthetic biology and nanotechnology continue to advance, the design of more precise, safe, and efficient microbial engineering strategies and nanobiomaterials is anticipated. These innovations are expected to establish a truly “microbe-driven” diagnostic and therapeutic paradigm in personalized tumor treatment plans. When synergistically applied with existing methods, such as immunotherapy, molecular-targeted therapy, and gene-editing strategies, these approaches could revolutionize patient care, rendering tumor microbiology an indispensable component in the era of precision medicine. However, despite the significant potential of integrating nanotechnology and microbiology into tumor therapy, a comprehensive understanding of the tumor microbiome remains essential. As emphasized in this article, a thorough characterization of the tumor microbiome and an in-depth exploration of its origins and mechanisms of influence on tumors will facilitate the advancement and application of cutting-edge fields, including nanotechnology. This not only promises the development of more specific and efficacious nanotherapeutic tumor treatment systems but also provides a more robust foundation for precision medicine.

## 8. Translational Challenges: From Mouse Models to Human Microbiome Research

### 8.1. Systematic Differences Between Mouse and Human Models

Mouse models constitute a fundamental experimental platform for microbiome oncology research, offering controlled environments for the mechanistic interrogation of microbiome–host interactions within tumor contexts. These systems provide exceptional opportunities to establish causal relationships between specific microbial taxa and critical oncological processes, including carcinogenesis, immunosurveillance modulation, and therapeutic response profiles. However, intrinsic species-specific variations in microbial ecology and host physiology impose significant constraints on the translational validity of the findings derived from murine systems. Anatomical and physiological disparities between murine and human hosts substantially influence microbial colonization dynamics and ecological establishment. The murine gastrointestinal tract, lacking an appendix and characterized by accelerated transit kinetics and distinct pH gradients compared to human counterparts, creates fundamentally different microenvironmental conditions for microbial niche formation. These architectural distinctions not only affect colonization patterns but also modulate microbial metabolic activities and host–microbe interface interactions. Furthermore, the standardized laboratory dietary regimens and controlled environmental exposures of laboratory mice contrast sharply with the heterogeneous dietary compositions and lifestyle factors that shape human microbiome development, complicating the cross-species extrapolation of findings.

At the taxonomic level, while murine and human intestinal microbiota exhibit considerable convergence at higher taxonomic classifications (notably the predominance of *Firmicutes* and *Bacteroidetes* phyla) [179,180], they diverge substantially at higher-resolution taxonomic levels. Despite an approximately 62% overlap at the genus level, merely 10% of bacterial species demonstrate conservation between these host systems [179]. This pronounced species-level divergence underscores the limitations inherent in modeling human-specific microbial functionalities, such as the production of immunomodulatory molecules, which frequently exhibit strain-dependent characteristics. These disparities extend beyond the intestinal environment; the murine vaginal microbiome notably lacks the *Lactobacillus* dominance characteristic of human vaginal homeostasis [62]. In the context of tumor microbiomes, spatial organization introduces additional layers of complexity. Human solid tumors harbor bacterial communities within hypoxic regions, necrotic cores, and immune cell-infiltrated areas, where the spatial gradients of metabolites and host–microbe signaling networks significantly influence therapeutic responsiveness. However, recapitulating these spatial dynamics of human tumor microbiota in murine models remains technically challenging. Nevertheless, emerging evidence suggests conservation of specific microbial signatures, including certain fungal communities and bacterial genera such as *Enterococcus* and *Streptococcus* between human and murine breast cancer models [29]. These inter-species conserved microbial signatures may illuminate common mechanistic pathways through which the tumor microbiome influences oncogenesis and disease progression, although translating these insights into human therapeutic applications remains complex.

### 8.2. Translational Advances from Preclinical Research to the Clinic

Mouse models remain indispensable tools in preclinical oncology research, providing critical mechanistic insights into tumorigenesis, disease progression, and treatment response within controlled experimental parameters, which can elucidate fundamental aspects of microbiome–tumor microenvironment interactions, particularly regarding the microbial influence on immune regulatory networks, therapeutic efficacy, and microbiota dynamics. Clinical investigations, conversely, have leveraged direct analyses of patient-derived specimens, including tumor tissue, peripheral blood, and fecal samples, to characterize microbial diversity, compositional alterations, and spatial distribution patterns within neoplastic tissues. Advanced molecular profiling approaches, including metagenomic shotgun sequencing and single-cell RNA analysis, have revealed distinctive microbial signatures associated with specific tumor molecular subtypes, metastatic potential, and clinical outcomes.

Contemporary research designs increasingly emphasize the integration of preclinical models and clinical trials to advance therapeutic development. In a notable example, preclinical investigations identified *Enterococcus hirae* and *Barnesiella intestinihominis* as enhancers of cyclophosphamide efficacy through distinct immunological mechanisms. A subsequent clinical evaluation in 38 patients with advanced malignancies demonstrated that individuals developing robust memory Th1 immune responses against these bacterial species experienced prolonged progression-free survival during chemotherapeutic intervention, effectively validating the conceptual framework established in preclinical systems [181]. Similarly, another investigation identified a tail length tape measure protein (TMP) from *E. hirae* in murine models that induces T cell responses cross-reactive with tumor antigens, thereby potentiating immunotherapeutic efficacy. These findings translated successfully to human studies, where cancer patients harboring TMP-encoding phages in their gut microbiota exhibited superior survival outcomes during PD-1 blockade therapy, and analogous cross-reactive T cell populations were detected in melanoma patients [182]. While these parallel investigations demonstrate successful translational trajectories, human studies typically provide correlative rather than causal evidence, reflecting inherent challenges in extrapolating mechanistic insights from murine systems to clinical contexts. Mechanistic preclinical investigations therefore require complementary clinical observational studies to establish causality and translational validity. For instance, despite murine studies suggesting certain microorganisms accelerate neoplastic development, investigations lacking definitive proof of direct microbial causality in oncogenesis may inadequately reflect human carcinogenic processes and require validation through human observational studies [183].

In the realm of bacterially mediated anti-tumor therapy, recent years have witnessed the emergence of clinical trials exploring therapeutic applications [184,185,186]. Advances in genetic engineering and nanotechnology continue to expand the potential of bacteriotherapy. Genetically modified bacterial strains, such as attenuated *Salmonella* for tumor-targeted interventions or *E. coli* engineered to express cytotoxic proteins, have demonstrated considerable promise in preclinical models by exploiting tumor-specific microenvironmental characteristics, particularly hypoxic regions [160,187]. Additionally, nanotechnological approaches, through surface modification or encapsulation techniques, enable the precise delivery of bacterial therapeutics or microbial metabolites in preclinical systems [159]. However, clinical experimental design needs to account for fundamental structural and biological differences between preclinical animal models and human subjects. Human tumors exhibit substantially greater architectural complexity and microenvironmental heterogeneity compared to experimental animal models [188]. While hypoxic and avascular tumor regions may provide ideal niches for bacterial colonization, interspecies differences in immune surveillance and clearance mechanisms may result in the premature elimination of bacterial therapeutics in clinical applications, potentially compromising therapeutic efficacy. Some investigators have implemented reverse translational methodologies, wherein clinical observations inform murine experimental design, thereby validating cross-species research findings. A recent investigation identified an enrichment of butyrate-producing bacteria in tumor specimens from lung cancer patients experiencing early recurrence [189]. This clinical observation subsequently guided mechanistic studies employing multiple murine models, including subcutaneous xenografts, orthotopic lung cancer models, and experimental metastasis assays, to elucidate mechanisms by which butyrate-producing bacteria and their metabolic product butyric acid promote pulmonary carcinoma metastasis. This bidirectional approach, initiated by human data and testing mechanistic hypotheses in animal models, provides a robust framework for cross-species validation. Nevertheless, these findings remain preliminary; definitive clinical relevance awaits confirmation through large-scale prospective cohort studies and interventional clinical trials.

### 8.3. Optimizing Preclinical Model Strategies: The Application of Novel Technological Platforms and Integrative Approaches

The translational efficacy of microbiome oncology research fundamentally depends on preclinical models, though their inherent limitations necessitate rigorous critical assessment. Contemporary model systems, encompassing genetically engineered mouse models (GEMMs), patient-derived xenograft (PDX) platforms, and humanized murine systems, have substantially advanced our mechanistic understanding of host–microbiota interactions within oncological contexts, yet significant constraints persist. GEMMs, engineered to recapitulate specific genetic aberrations characteristic of human malignancies, offer valuable insights into microbiome–cancer interactions. The KPC mouse model (LSL-Kras^G12D/+^; LSL-Trp53^R172H/+^; Pdx-1-Cre), which faithfully reproduces the clinicopathological and microbiological features of human pancreatic ductal adenocarcinoma, demonstrates a significantly enriched and distinctive bacterial community within pancreatic neoplastic tissue compared to healthy pancreatic parenchyma. Notably, the tumor-associated microbiota in KPC mice exhibits similarity to that observed in human PDAC specimens [30]. However, this concordance is highly context-dependent; without explicit humanization procedures, the intestinal microbiota in GEMMs remains predominantly murine, underscoring the necessity for judicious model selection and interpretation. Similarly, PDX models, wherein human neoplastic cells are engrafted into immunocompromised murine hosts, are extensively employed in preclinical investigations including pharmacological screening, the elucidation of drug resistance mechanisms, the development of targeted therapeutic modalities, and the evaluation of immunotherapeutic agents [190,191]. Nevertheless, these models fundamentally alter microbiome–immune system interactions, constraining their capacity to accurately recapitulate human physiological milieus. Recent advances in humanized microbiota mouse models, involving the transplantation of human microbiota into germ-free murine hosts, have demonstrated promising translational potential. A novel humanized (THX) mouse model has recently been developed wherein genetically modified immune-deficient mice are engrafted with human umbilical cord blood stem cells and conditioned with 17β-estradiol, resulting in the development of a functionally competent human-like immune system [192]. Critically, the intestinal microbiota of THX mice demonstrates greater similarity to human intestinal microbiomes, sharing bacterial families with human intestinal communities and exhibiting significant divergence from conventional NBSGW murine microbiomes. This model represents a substantial advancement in translational research, particularly for immunological investigations including autoimmune responses and vaccine development. While these innovative platforms constitute significant progress, their utility requires validation across diverse oncological contexts, and fundamental questions persist regarding their capacity to fully capture the co-evolutionary dynamics between human hosts and their associated microbiota, particularly within specialized tissue microenvironments such as neoplastic lesions. Concurrent refinements in microbiome detection methodologies further influence translational validity. A recently proposed optimized analytical framework integrates highly sensitive quantitative PCR technology for precise bacterial enumeration with 16S amplicon sequencing for comprehensive microbial diversity assessment, enabling the accurate quantification and profiling of tissue-resident microbiomes in both human and murine specimens [40]. Such technological advancements are indispensable for ensuring robust cross-species comparability.

To circumvent the translational limitations inherent in single-model experimental systems, investigators increasingly implement multi-platform frameworks. Microfluidic chip technology possesses transformative potential in oncological research by providing physiologically relevant experimental platforms surpassing traditional in vitro and animal systems. This technological approach demonstrates exceptional promise for personalized medical strategies and pharmaceutical development optimization through its capacity to recapitulate complex tissue–tissue interfaces, mechanical forces, and fluidic dynamic characteristics of living organs, while simultaneously enabling the precise manipulation of cellular, molecular, and biophysical parameters [193]. A recent investigation developed a human intestinal chip model incorporating human fecal microbiome components and peristaltic-like mechanical stimulation to simulate microbiome–host interactions and predict immunological responses to melanoma immunotherapeutics. This model effectively replicates the human intestinal environment and provides a valuable platform for investigating microbiome-mediated effects on immunotherapeutic responsiveness [194]. This innovative research strategy substantially enhances the reliability and translational efficacy of preclinical findings. Additionally, systematic validation through multi-omics technological approaches constitutes a critical element for augmenting the translational value of experimental models. With the continuous refinement of detection methodologies and the development of novel research platforms, this integrated approach will progressively advance microbiome oncology research toward enhanced translational significance and clinical applicability.

## 9. Conclusions

Research on the tumor microbiome has emerged as a transformative approach within the field of cancer studies, unveiling its pivotal roles in tumor biology and therapeutic efficacy. Resident microorganisms within tumor tissues actively interact with cancer cells, immune components, and the surrounding microenvironment, thereby influencing processes such as tumor progression, immune modulation, and drug resistance. Compared to the gut microbiome, the tumor-associated microbiome exhibits unique complexity, often reflecting the specific characteristics of the tumor and demonstrating significant heterogeneity across different types of cancer. These findings underscore the potential of the tumor microbiome as biomarkers for cancer diagnosis and prognostic evaluation, as well as novel targets for therapeutic interventions. Despite substantial progress, numerous challenges remain, including elucidating the origins of tumor-associated microbes, understanding their functional adaptations within the harsh TME, and deciphering the precise mechanisms of their interactions with host pathways. Addressing these challenges necessitates a multidisciplinary approach that integrates advancements in microbiology, cancer biology, and bioinformatics with innovative tools such as single-cell and spatial omics technologies. Additionally, ensuring rigorous experimental design and data interpretation is crucial for overcoming issues related to contamination and enhancing reproducibility. Looking ahead, the therapeutic potential of the tumor microbiome is particularly noteworthy. Strategies for selectively modulating these microbial communities, whether through direct regulation or engineered bacterial therapies, have the capacity to complement existing treatment modalities and enhance their efficacy. When integrated within the framework of precision medicine, these approaches hold promise for achieving personalized therapies based on the microbial composition of individual tumors, thereby fundamentally transforming cancer treatment paradigms. As the field continues to advance, the integration of these insights into clinical practice will depend on robust validation through preclinical and clinical studies. In summary, research on the tumor microbiome is redefining our understanding of tumor biology and paving the way for innovative therapeutic strategies. By addressing existing challenges and leveraging emerging technologies, this rapidly evolving field holds immense promise for advancing cancer diagnosis and treatment, ultimately improving patient prognoses.

## Figures and Tables

**Table 1:** Bacterial contributions to cancer: types, effects, and mechanisms.

Cancer Type	Sample Source	Sequencing/Detection Method	Bacteria Compositions	Effect	Mechanism
Colorectal Cancer	Human colon specimens	16S rRNA sequencing	*F. nucleatum, Providencia* [34]	Promoting tumor progression	FadA binds E-cadherin and activates β-catenin, inducing inflammation and proliferation [35] Fap2 binds CEACAM1 on immune cells, inhibiting NK and T cell anti-tumor activity for immune evasion [36,37]
		BFT polymerase chain reaction analysis	*Bacteroides fragilis* [38]	Promoting tumor progression	BFT disrupts tight junctions, activates Wnt/β-catenin, and induces pro-inflammatory cytokines [38,39]
		An optimized protocol of qPCR and 16S sequencing [40]	*E*. *coli* [41]	Initiation of cancers	Colibactin-producing *E. coli* (pks+) induce DNA cross-linking, causing DNA damage and mutations [42]
Breast Cancer	Human breast tumor specimens	PathoChip microarray with genome/transcriptome amplification	*E. coli* [43,44]	Promoting tumor progression	*E. coli*’s N-acetyl-L-methionine generates methionine and acetate for glutathione synthesis, scavenging ROS and promoting cancer growth [45]
		16S rRNA sequencing	*Bacillus* [44]	Promoting tumor progression	Converts progesterone to 5αP [44,46]
				Inhibited proliferation of certain breast cancer cells (T47D, MDA-MB-468, HCC1428, MDA-MB-453)	EPS (an exopolysaccharide produced by *Bacillus subtilis*) activates an inflammatory response in sensitive breast cancer cells through activation of TNF, interferon/JAK-STAT, and/or NF-κB signaling [47]
			*Staphylococcus* [44]	Promoting tumor progression	DNA damage, activation of the ataxia–telangiectasia mutated (ATM)–p53 signaling pathway [48]
	Mouse breast tumors	An optimized protocol of qPCR and 16S sequencing	*Lactobacillus* [29]	Enhancing immunotherapy efficacy	Metabolite indole-3-propionic acid (IPA) enhances the efficacy of CD8 T cell-mediated αPD-1 immunotherapy [49]
				Promoting tumor progression	Suppresses the anti-tumor activities of immune cells, particularly CD8T cells, and promotes polarization of M2 macrophages [50]
			*Staphylococcus* [29]	Promoting tumor progression	Creates a sustained inflammatory environment and break DNA, promoting cellular proliferation, angiogenesis, and genetic instability [51]
			*Enterococcus* [29]	Unclear and controversial	Unclear [52,53]
Lung Cancer	Human tumor samples	Culturomics and 16S rRNA sequencing	*Streptococcus* [54]	Promoting tumor progression	Activates monocytes, increases IL-6, IL-12, and TNF, promoting Th1 and Th17 inflammation [55]. Activates PI3K/AKT and NF-kB pathways [56]
			*Veilonella* [54]	Promoting tumor progression	Unclear [57,58]
Pancreatic Ductal Adenocarcinoma	Human tumor specimens	16S rRNA sequencing	*Proteobacteria, Bacteroidetes*, *Firmicutes* [31]	Promoting tumor progression	Promoting tumor progression [31]
			*Sachharopolyspora*, *Pseudoxanthomas*, *Streptomyces* [59]	Enhancing anti-tumor immune response	Anti-CTLA4 and anti-PD-L1 [59]
	Mouse pancreas	16S rRNA sequencing	*Agrobacterium*, *Rhizobium* [31]	Unclear	Unclear
Ovarian Cancer	Human ovarian cancer specimens	16S rRNA sequencing	*Proteobacteria*, *Firmicutes*, *Acinetobacter* [60]	Promoting tumor progression	Influence “JAK-STAT signaling pathway”, “transcriptional misregulation in cancer”, and “Th1 and Th2 cell differentiation” pathway [61]
			*Lactococcus* [60]	Enhancing anti-tumor immune response	Unclear
	Mouse oviducts, ovaries	16S rRNA sequencing	*Bacteroidales*, *Clostridium*, *Blautia*, *Lachnospiraceae* [62]	Promoting tumor progression	Unclear

## Data Availability

Not applicable.

## Abbreviations

The following abbreviations are used in this manuscript:

TME	tumor microenvironment
ECM	extracellular matrix
TAMs	tumor-associated macrophages
Tregs	regulatory T cells
MDSCs	myeloid-derived suppressor cells
FISH	fluorescence in situ hybridization
ATM	Ataxia–telangiectasia mutated
IPA	indole-3-propionic acid
OSCC	oral squamous cell carcinoma
SCFA	short-chain fatty acid
HIF	hypoxia-inducible factor
LPS	lipopolysaccharide
IS-pro	Intergenic Spacer Profiling
IHC	immunohistochemistry
HRP	Horseradish Peroxidase
CLEM	correlative light and electron microscopy
CLSM	confocal laser scanning microscopy
EM	electron microscopy
TEM	transmission electron microscopy
PDOs	patient-derived organoids
CRC	colorectal cancer
ETBF	enterotoxigenic Bacteroides fragilis
PMNs	pre-metastatic niches
DCA	deoxycholic acid
5-FU	5-fluorouracil
EMT	epithelial-to-mesenchymal transition
ICIs	immune checkpoint inhibitors
IL-17	interleukin-17
PDAC	pancreatic ductal adenocarcinoma
LTS	long-term survivors
STS	short-term survivors
NSCLC	non-small-cell lung cancer
LUAD	lung adenocarcinoma
OMVs	outer membrane vesicles
TMP	tail-length tape-measure protein
GEMMs	genetically engineered mouse models
PDX	patient-derived xenograft

## References

[1] Iliev I.D., Cadwell K. (2021). Effects of Intestinal Fungi and Viruses on Immune Responses and Inflammatory Bowel Diseases. Gastroenterology.

[2] Chen J., Domingue J.C., Sears C.L. (2017). Microbiota dysbiosis in select human cancers: Evidence of association and causality. Semin. Immunol..

[3] Zhao L., Luo J.L., Ali M.K., Spiekerkoetter E., Nicolls M.R. (2023). The Human Respiratory Microbiome: Current Understandings and Future Directions. Am. J. Respir. Cell Mol. Biol..

[4] Meng S., Chen B., Yang J., Wang J., Zhu D., Meng Q., Zhang L. (2018). Study of Microbiomes in Aseptically Collected Samples of Human Breast Tissue Using Needle Biopsy and the Potential Role of in situ Tissue Microbiomes for Promoting Malignancy. Front. Oncol..

[5] Nejman D., Livyatan I., Fuks G., Gavert N., Zwang Y., Geller L.T., Rotter-Maskowitz A., Weiser R., Mallel G., Gigi E. (2020). The human tumor microbiome is composed of tumor type-specific intracellular bacteria. Science.

[6] Xie Y., Xie F., Zhou X., Zhang L., Yang B., Huang J., Wang F., Yan H., Zeng L., Zhang L. (2022). Microbiota in Tumors: From Understanding to Application. Adv. Sci..

[7] Chen F., Yang J., Guo Y., Su D., Sheng Y., Wu Y. (2023). Integrating bulk and single-cell RNA sequencing data reveals the relationship between intratumor microbiome signature and host metabolic heterogeneity in breast cancer. Front. Immunol..

[8] Laborda-Illanes A., Sanchez-Alcoholado L., Dominguez-Recio M.E., Jimenez-Rodriguez B., Lavado R., Comino-Mendez I., Alba E., Queipo-Ortuno M.I. (2020). Breast and Gut Microbiota Action Mechanisms in Breast Cancer Pathogenesis and Treatment. Cancers.

[9] Li J., Guo Y., Liu J., Guo F., Du L., Yang Y., Li X., Ma Y. (2023). Depicting the landscape of gut microbial-metabolic interaction and microbial-host immune heterogeneity in deficient and proficient DNA mismatch repair colorectal cancers. J. Immunother. Cancer.

[10] Amatya S.B., Salmi S., Kainulainen V., Karihtala P., Reunanen J. (2021). Bacterial Extracellular Vesicles in Gastrointestinal Tract Cancer: An Unexplored Territory. Cancers.

[11] Zhao K., Hu Y. (2020). Microbiome harbored within tumors: A new chance to revisit our understanding of cancer pathogenesis and treatment. Signal Transduct. Target. Ther..

[12] Ciernikova S., Sevcikova A., Stevurkova V., Mego M. (2022). Tumor microbiome—An integral part of the tumor microenvironment. Front. Oncol..

[13] Liu Z., Zhang X., Zhang H., Zhang H., Yi Z., Zhang Q., Liu Q., Liu X. (2023). Multi-Omics Analysis Reveals Intratumor Microbes as Immunomodulators in Colorectal Cancer. Microbiol. Spectr..

[14] Wang Z.H., Chu M., Yin N., Huang W., Liu W., Zhang Z., Liu J., Shi J. (2022). Biological chemotaxis-guided self-thermophoretic nanoplatform augments colorectal cancer therapy through autonomous mucus penetration. Sci. Adv..

[15] Mittal R., Patel A.P., Jhaveri V.M., Kay S.S., Debs L.H., Parrish J.M., Pan D.R., Nguyen D., Mittal J., Jayant R.D. (2018). Recent advancements in nanoparticle based drug delivery for gastrointestinal disorders. Expert Opin. Drug Deliv..

[16] Truffi M., Sorrentino L., Corsi F. (2020). Fibroblasts in the Tumor Microenvironment. Adv. Exp. Med. Biol..

[17] Anderson N.M., Simon M.C. (2020). The tumor microenvironment. Curr. Biol..

[18] Sounni N.E., Noel A. (2013). Targeting the tumor microenvironment for cancer therapy. Clin. Chem..

[19] Li M.O., Wolf N., Raulet D.H., Akkari L., Pittet M.J., Rodriguez P.C., Kaplan R.N., Munitz A., Zhang Z., Cheng S. (2021). Innate immune cells in the tumor microenvironment. Cancer Cell.

[20] Hanahan D., Weinberg R.A. (2011). Hallmarks of cancer: The next generation. Cell.

[21] Roma-Rodrigues C., Mendes R., Baptista P.V., Fernandes A.R. (2019). Targeting Tumor Microenvironment for Cancer Therapy. Int. J. Mol. Sci..

[22] Pitt J.M., Marabelle A., Eggermont A., Soria J.C., Kroemer G., Zitvogel L. (2016). Targeting the tumor microenvironment: Removing obstruction to anticancer immune responses and immunotherapy. Ann. Oncol..

[23] Roy S., Trinchieri G. (2017). Microbiota: A key orchestrator of cancer therapy. Nat. Rev. Cancer.

[24] Oliva M., Mulet-Margalef N., Ochoa-De-Olza M., Napoli S., Mas J., Laquente B., Alemany L., Duell E.J., Nuciforo P., Moreno V. (2021). Tumor-Associated Microbiome: Where Do We Stand?. Int. J. Mol. Sci..

[25] Dudgeon L.S., Dunkley E.V. (1907). The Micrococcus neoformans: Its Cultural Characters and Pathogenicity and the Results of the Estimation of the Opsonic and Agglutinative Properties of the Serum of Patients Suffering from Malignant Disease on this Organism and on the Staphylococcus Albus. J. Hyg..

[26] Amieva M., Peek R.M. (2016). Pathobiology of Helicobacter pylori-Induced Gastric Cancer. Gastroenterology.

[27] Tjalsma H., Boleij A., Marchesi J.R., Dutilh B.E. (2012). A bacterial driver-passenger model for colorectal cancer: Beyond the usual suspects. Nat. Rev. Microbiol..

[28] Kostic A.D., Chun E., Robertson L., Glickman J.N., Gallini C.A., Michaud M., Clancy T.E., Chung D.C., Lochhead P., Hold G.L. (2013). Fusobacterium nucleatum potentiates intestinal tumorigenesis and modulates the tumor-immune microenvironment. Cell Host Microbe.

[29] Fu A., Yao B., Dong T., Chen Y., Yao J., Liu Y., Li H., Bai H., Liu X., Zhang Y. (2022). Tumor-resident intracellular microbiota promotes metastatic colonization in breast cancer. Cell.

[30] Pfisterer N., Ammer-Herrmenau C., Antweiler K., Kuffer S., Ellenrieder V., Neesse A. (2023). Dynamics of intestinal and intratumoral microbiome signatures in genetically engineered mice and human pancreatic ductal adenocarcinoma. Pancreatology.

[31] Pushalkar S., Hundeyin M., Daley D., Zambirinis C.P., Kurz E., Mishra A., Mohan N., Aykut B., Usyk M., Torres L.E. (2018). The Pancreatic Cancer Microbiome Promotes Oncogenesis by Induction of Innate and Adaptive Immune Suppression. Cancer Discov..

[32] Rubiola S., Macori G., Civera T., Fanning S., Mitchell M., Chiesa F. (2022). Comparison Between Full-Length 16S rRNA Metabarcoding and Whole Metagenome Sequencing Suggests the Use of Either Is Suitable for Large-Scale Microbiome Studies. Foodborne Pathog. Dis..

[33] Biegert G., El Alam M.B., Karpinets T., Wu X., Sims T.T., Yoshida-Court K., Lynn E.J., Yue J., Medrano A.D., Petrosino J. (2021). Diversity and composition of gut microbiome of cervical cancer patients: Do results of 16S rRNA sequencing and whole genome sequencing approaches align?. J. Microbiol. Methods.

[34] Burns M.B., Lynch J., Starr T.K., Knights D., Blekhman R. (2015). Virulence genes are a signature of the microbiome in the colorectal tumor microenvironment. Genome Med..

[35] Rubinstein M.R., Wang X., Liu W., Hao Y., Cai G., Han Y.W. (2013). Fusobacterium nucleatum promotes colorectal carcinogenesis by modulating E-cadherin/β-catenin signaling via its FadA adhesin. Cell Host Microbe.

[36] Brewer M.L., Dymock D., Brady R.L., Singer B.B., Virji M., Hill D.J. (2019). Fusobacterium spp. target human CEACAM1 via the trimeric autotransporter adhesin CbpF. J. Oral Microbiol..

[37] Galaski J., Shhadeh A., Umana A., Yoo C.C., Arpinati L., Isaacson B., Berhani O., Singer B.B., Slade D.J., Bachrach G. (2021). Fusobacterium nucleatum CbpF Mediates Inhibition of T Cell Function Through CEACAM1 Activation. Front. Cell Infect. Microbiol..

[38] Boleij A., Hechenbleikner E.M., Goodwin A.C., Badani R., Stein E.M., Lazarev M.G., Ellis B., Carroll K.C., Albesiano E., Wick E.C. (2015). The Bacteroides fragilis toxin gene is prevalent in the colon mucosa of colorectal cancer patients. Clin. Infect. Dis..

[39] Sears C.L., Geis A.L., Housseau F. (2014). Bacteroides fragilis subverts mucosal biology: From symbiont to colon carcinogenesis. J. Clin. Investig..

[40] Yao B., Dong T., Fu A., Li H., Jiang C., Li N., Cai S. (2022). Quantification and characterization of mouse and human tissue-resident microbiota by qPCR and 16S sequencing. STAR Protoc..

[41] Gu J., Xu X., Li X., Yue L., Zhu X., Chen Q., Gao J., Takashi M., Zhao W., Zhao B. (2024). Tumor-resident microbiota contributes to colorectal cancer liver metastasis by lactylation and immune modulation. Oncogene.

[42] Arthur J.C., Perez-Chanona E., Muhlbauer M., Tomkovich S., Uronis J.M., Fan T.J., Campbell B.J., Abujamel T., Dogan B., Rogers A.B. (2012). Intestinal inflammation targets cancer-inducing activity of the microbiota. Science.

[43] Banerjee S., Tian T., Wei Z., Shih N., Feldman M.D., Peck K.N., DeMichele A.M., Alwine J.C., Robertson E.S. (2018). Distinct Microbial Signatures Associated with Different Breast Cancer Types. Front. Microbiol..

[44] Urbaniak C., Gloor G.B., Brackstone M., Scott L., Tangney M., Reid G. (2016). The Microbiota of Breast Tissue and Its Association with Breast Cancer. Appl. Environ. Microbiol..

[45] AlMalki R.H., Sebaa R., Al-Ansari M.M., Al-Alwan M., Alwehaibi M.A., Rahman A.M.A.E. (2023). coli Secretome Metabolically Modulates MDA-MB-231 Breast Cancer Cells’ Energy Metabolism. Int. J. Mol. Sci..

[46] Wiebe J.P., Muzia D., Hu J., Szwajcer D., Hill S.A., Seachrist J.L. (2000). The 4-pregnene and 5alpha-pregnane progesterone metabolites formed in nontumorous and tumorous breast tissue have opposite effects on breast cell proliferation and adhesion. Cancer Res..

[47] Nguyen M.R., Ma E., Wyatt D., Knight K.L., Osipo C. (2023). The effect of an exopolysaccharide probiotic molecule from Bacillus subtilis on breast cancer cells. Front. Oncol..

[48] Zhao H., Zhang L., Du D., Mai L., Liu Y., Morigen M., Fan L. (2024). The RIG-I-like receptor signaling pathway triggered by Staphylococcus aureus promotes breast cancer metastasis. Int. Immunopharmacol..

[49] Jia D., Wang Q., Qi Y., Jiang Y., He J., Lin Y., Sun Y., Xu J., Chen W., Fan L. (2024). Microbial metabolite enhances immunotherapy efficacy by modulating T cell stemness in pan-cancer. Cell.

[50] Shi Q., Wang J., Zhou M., Zheng R., Zhang X., Liu B. (2023). Gut Lactobacillus contribute to the progression of breast cancer by affecting the anti-tumor activities of immune cells in the TME of tumor-bearing mice. Int. Immunopharmacol..

[51] Allen-Taylor D., Boro G., Cabato P.M., Mai C., Nguyen K., Rijal G. (2024). Staphylococcus epidermidis biofilm in inflammatory breast cancer and its treatment strategies. Biofilm.

[52] Cardeiro M., Ardeljan A.D., Frankel L., Kim E., Takabe K., Rashid O.M. (2023). Incidence of Breast Cancer and Enterococcus Infection: A Retrospective Analysis. World J. Oncol..

[53] Kim H.E., Kim J., Maeng S., Oh B., Hwang K.T., Kim B.S. (2021). Microbiota of Breast Tissue and Its Potential Association with Regional Recurrence of Breast Cancer in Korean Women. J. Microbiol. Biotechnol..

[54] Sun Y., Liu Y., Li J., Tan Y., An T., Zhuo M., Pan Z., Ma M., Jia B., Zhang H. (2023). Characterization of Lung and Oral Microbiomes in Lung Cancer Patients Using Culturomics and 16S rRNA Gene Sequencing. Microbiol. Spectr..

[55] Ma Q.Y., Huang D.Y., Zhang H.J., Wang S., Chen X.F. (2017). Upregulation of bacterial-specific Th1 and Th17 responses that are enriched in CXCR5(+)CD4(+) T cells in non-small cell lung cancer. Int. Immunopharmacol..

[56] Li N., Zhou H., Holden V.K., Deepak J., Dhilipkannah P., Todd N.W., Stass S.A., Jiang F. (2023). Streptococcus pneumoniae promotes lung cancer development and progression. iScience.

[57] Yu G., Gail M.H., Consonni D., Carugno M., Humphrys M., Pesatori A.C., Caporaso N.E., Goedert J.J., Ravel J., Landi M.T. (2016). Characterizing human lung tissue microbiota and its relationship to epidemiological and clinical features. Genome Biol..

[58] Karvela A., Veloudiou O.Z., Karachaliou A., Kloukina T., Gomatou G., Kotteas E. (2023). Lung microbiome: An emerging player in lung cancer pathogenesis and progression. Clin. Transl. Oncol..

[59] Riquelme E., Zhang Y., Zhang L., Montiel M., Zoltan M., Dong W., Quesada P., Sahin I., Chandra V., San Lucas A. (2019). Tumor Microbiome Diversity and Composition Influence Pancreatic Cancer Outcomes. Cell.

[60] Zhou B., Sun C., Huang J., Xia M., Guo E., Li N., Lu H., Shan W., Wu Y., Li Y. (2019). The biodiversity Composition of Microbiome in Ovarian Carcinoma Patients. Sci. Rep..

[61] Li M., He G., Kong F., Wang P., Han C., Ding Q., Jiang H., Deng S. (2024). Unraveling the role of tissue colonized microbiome in ovarian cancer progression. Comput. Biol. Med..

[62] Chen L., Zhai Y., Wang Y., Fearon E.R., Nunez G., Inohara N., Cho K.R. (2021). Altering the Microbiome Inhibits Tumorigenesis in a Mouse Model of Oviductal High-Grade Serous Carcinoma. Cancer Res..

[63] Zhang Z., Feng Q., Li M., Li Z., Xu Q., Pan X., Chen W. (2022). Age-Related Cancer-Associated Microbiota Potentially Promotes Oral Squamous Cell Cancer Tumorigenesis by Distinct Mechanisms. Front. Microbiol..

[64] Tomkovich S., Yang Y., Winglee K., Gauthier J., Muhlbauer M., Sun X., Mohamadzadeh M., Liu X., Martin P., Wang G.P. (2017). Locoregional Effects of Microbiota in a Preclinical Model of Colon Carcinogenesis. Cancer Res..

[65] Biragyn A., Ferrucci L. (2018). Gut dysbiosis: A potential link between increased cancer risk in ageing and inflammaging. Lancet Oncol..

[66] Kelly C.J., Zheng L., Campbell E.L., Saeedi B., Scholz C.C., Bayless A.J., Wilson K.E., Glover L.E., Kominsky D.J., Magnuson A. (2015). Crosstalk between Microbiota-Derived Short-Chain Fatty Acids and Intestinal Epithelial HIF Augments Tissue Barrier Function. Cell Host Microbe.

[67] Minich J.J., Humphrey G., Benitez R.A.S., Sanders J., Swafford A., Allen E.E., Knight R. (2018). High-Throughput Miniaturized 16S rRNA Amplicon Library Preparation Reduces Costs while Preserving Microbiome Integrity. mSystems.

[68] De Muinck E.J., Trosvik P., Gilfillan G.D., Hov J.R., Sundaram A.Y.M. (2017). A novel ultra high-throughput 16S rRNA gene amplicon sequencing library preparation method for the Illumina HiSeq platform. Microbiome.

[69] Perez-Cobas A.E., Gomez-Valero L., Buchrieser C. (2020). Metagenomic approaches in microbial ecology: An update on whole-genome and marker gene sequencing analyses. Microb. Genom..

[70] Huang X., Chen C., Xie W., Zhou C., Tian X., Zhang Z., Wang Q., Chang H., Xiao W., Zhang R. (2023). Metagenomic Analysis of Intratumoral Microbiome Linking to Response to Neoadjuvant Chemoradiotherapy in Rectal Cancer. Int. J. Radiat. Oncol. Biol. Phys..

[71] Taj B., Adeolu M., Xiong X., Ang J., Nursimulu N., Parkinson J. (2023). MetaPro: A scalable and reproducible data processing and analysis pipeline for metatranscriptomic investigation of microbial communities. Microbiome.

[72] Budding A.E., Grasman M.E., Lin F., Bogaards J.A., Soeltan-Kaersenhout D.J., Vandenbroucke-Grauls C.M., van Bodegraven A.A., Savelkoul P.H. (2010). IS-pro: High-throughput molecular fingerprinting of the intestinal microbiota. FASEB J..

[73] Qian X., Zhang H.Y., Li Q.L., Ma G.J., Chen Z., Ji X.M., Li C.Y., Zhang A.Q. (2022). Integrated microbiome, metabolome, and proteome analysis identifies a novel interplay among commensal bacteria, metabolites and candidate targets in non-small cell lung cancer. Clin. Transl. Med..

[74] Cai M., Kandalai S., Tang X., Zheng Q. (2022). Contributions of Human-Associated Archaeal Metabolites to Tumor Microenvironment and Carcinogenesis. Microbiol. Spectr..

[75] Ray D.M., Jennings E.Q., Maksimovic I., Chai X., Galligan J.J., David Y., Zheng Q. (2022). Chemical Labeling and Enrichment of Histone Glyoxal Adducts. ACS Chem. Biol..

[76] Allis C.D., Jenuwein T. (2016). The molecular hallmarks of epigenetic control. Nat. Rev. Genet..

[77] Tan W.C.C., Nerurkar S.N., Cai H.Y., Ng H.H.M., Wu D., Wee Y.T.F., Lim J.C.T., Yeong J., Lim T.K.H. (2020). Overview of multiplex immunohistochemistry/immunofluorescence techniques in the era of cancer immunotherapy. Cancer Commun..

[78] Balciuniene J., Ning Y., Lazarus H.M., Aikawa V., Sherpa S., Zhang Y., Morrissette J.J.D. (2024). Cancer cytogenetics in a genomics world: Wedding the old with the new. Blood Rev..

[79] Pope I., Tanner H., Masia F., Payne L., Arkill K.P., Mantell J., Langbein W., Borri P., Verkade P. (2023). Correlative light-electron microscopy using small gold nanoparticles as single probes. Light. Sci. Appl..

[80] Chai X., Wang J., Li H., Gao C., Li S., Wei C., Huang J., Tian Y., Yuan J., Lu J. (2023). Intratumor microbiome features reveal antitumor potentials of intrahepatic cholangiocarcinoma. Gut Microbes.

[81] Ghaddar B., Biswas A., Harris C., Omary M.B., Carpizo D.R., Blaser M.J., De S. (2022). Tumor microbiome links cellular programs and immunity in pancreatic cancer. Cancer Cell.

[82] Wuputra K., Ku C.C., Kato K., Wu D.C., Saito S., Yokoyama K.K. (2021). Translational models of 3-D organoids and cancer stem cells in gastric cancer research. Stem Cell Res. Ther..

[83] Mo S., Tang P., Luo W., Zhang L., Li Y., Hu X., Ma X., Chen Y., Bao Y., He X. (2022). Patient-Derived Organoids from Colorectal Cancer with Paired Liver Metastasis Reveal Tumor Heterogeneity and Predict Response to Chemotherapy. Adv. Sci..

[84] Aguilar C., Pauzuolis M., Pompaiah M., Vafadarnejad E., Arampatzi P., Fischer M., Narres D., Neyazi M., Kayisoglu O., Sell T. (2022). Helicobacter pylori shows tropism to gastric differentiated pit cells dependent on urea chemotaxis. Nat. Commun..

[85] Nagai T., Shiba T., Komatsu K., Watanabe T., Nemoto T., Maekawa S., Kobayashi R., Matsumura S., Ohsugi Y., Katagiri S. (2024). Optimal 16S rRNA gene amplicon sequencing analysis for oral microbiota to avoid the potential bias introduced by trimming length, primer, and database. Microbiol. Spectr..

[86] Sofie T., Michiel O.D.B., Bram B., Sascha T., Vincent S., Van H.J.D., Nele W., Jaco V. (2017). Comparative Evaluation of Four Bacteria-Specific Primer Pairs for 16S rRNA Gene Surveys. Front. Microbiol..

[87] Su X., Pan W., Song B., Xu J., Ning K. (2014). Parallel-META 2.0: Enhanced metagenomic data analysis with functional annotation, high performance computing and advanced visualization. PLoS ONE.

[88] Wooley J.C., Godzik A., Friedberg I. (2010). A primer on metagenomics. PLoS Comput. Biol..

[89] Ankney J.A., Muneer A., Chen X. (2018). Relative and Absolute Quantitation in Mass Spectrometry-Based Proteomics. Annu. Rev. Anal. Chem..

[90] Mann M., Kelleher N.L. (2008). Precision proteomics: The case for high resolution and high mass accuracy. Proc. Natl. Acad. Sci. USA.

[91] Cheison S.C., Kulozik U. (2017). Impact of the environmental conditions and substrate pre-treatment on whey protein hydrolysis: A review. Crit. Rev. Food Sci. Nutr..

[92] Krug S., Kastenmuller G., Stuckler F., Rist M.J., Skurk T., Sailer M., Raffler J., Romisch-Margl W., Adamski J., Prehn C. (2012). The dynamic range of the human metabolome revealed by challenges. FASEB J..

[93] Li Z., Li Y., Chen W., Cao Q., Guo Y., Wan N., Jiang X., Tang Y.J., Wang Q., Shui W. (2017). Integrating MS1 and MS2 Scans in High-Resolution Parallel Reaction Monitoring Assays for Targeted Metabolite Quantification and Dynamic (13)C-Labeling Metabolism Analysis. Anal. Chem..

[94] Poore G.D., Kopylova E., Zhu Q., Carpenter C., Fraraccio S., Wandro S., Kosciolek T., Janssen S., Metcalf J., Song S.J. (2020). RETRACTED ARTICLE: Microbiome analyses of blood and tissues suggest cancer diagnostic approach. Nature.

[95] Gihawi A., Ge Y., Lu J., Puiu D., Xu A., Cooper C.S., Brewer D.S., Pertea M., Salzberg S.L. (2023). Major data analysis errors invalidate cancer microbiome findings. mBio.

[96] Sepich-Poore G.D., McDonald D., Kopylova E., Guccione C., Zhu Q., Austin G., Carpenter C., Fraraccio S., Wandro S., Kosciolek T. (2024). Robustness of cancer microbiome signals over a broad range of methodological variation. Oncogene.

[97] Narunsky-Haziza L., Sepich-Poore G.D., Livyatan I., Asraf O., Martino C., Nejman D., Gavert N., Stajich J.E., Amit G., Gonzalez A. (2022). Pan-cancer analyses reveal cancer-type-specific fungal ecologies and bacteriome interactions. Cell.

[98] Huang J., Mao Y., Wang L. (2023). The crosstalk of intratumor bacteria and the tumor. Front. Cell Infect. Microbiol..

[99] Xu S., Xiong Y., Fu B., Guo D., Sha Z., Lin X., Wu H. (2023). Bacteria and macrophages in the tumor microenvironment. Front. Microbiol..

[100] Zhang Y., Zhang J., Xia Y., Sun J. (2023). Bacterial translocation and barrier dysfunction enhance colonic tumorigenesis. Neoplasia.

[101] Liu X., Cheng Y., Shao L., Ling Z. (2020). Alterations of the Predominant Fecal Microbiota and Disruption of the Gut Mucosal Barrier in Patients with Early-Stage Colorectal Cancer. BioMed Res. Int..

[102] Kordahi M.C., Stanaway I.B., Avril M., Chac D., Blanc M.P., Ross B., Diener C., Jain S., McCleary P., Parker A. (2021). Genomic and functional characterization of a mucosal symbiont involved in early-stage colorectal cancer. Cell Host Microbe.

[103] Fang J., Liao L., Yin H., Nakamura H., Shin T., Maeda H. (2014). Enhanced bacterial tumor delivery by modulating the EPR effect and therapeutic potential of Lactobacillus casei. J. Pharm. Sci..

[104] Bertocchi A., Carloni S., Ravenda P.S., Bertalot G., Spadoni I., Lo Cascio A., Gandini S., Lizier M., Braga D., Asnicar F. (2021). Gut vascular barrier impairment leads to intestinal bacteria dissemination and colorectal cancer metastasis to liver. Cancer Cell.

[105] Bullman S., Pedamallu C.S., Sicinska E., Clancy T.E., Zhang X., Cai D., Neuberg D., Huang K., Guevara F., Nelson T. (2017). Analysis of Fusobacterium persistence and antibiotic response in colorectal cancer. Science.

[106] Li Y., Zhao L., Li X.F. (2021). Hypoxia and the Tumor Microenvironment. Technol. Cancer Res. Treat..

[107] Chieppa M., Kashyrina M., Miraglia A., Vardanyan D. (2024). Enhanced CRC Growth in Iron-Rich Environment, Facts and Speculations. Int. J. Mol. Sci..

[108] Rajabi M., Mousa S.A. (2017). The Role of Angiogenesis in Cancer Treatment. Biomedicines.

[109] Weitzman M.D., Weitzman J.B. (2014). What’s the damage? The impact of pathogens on pathways that maintain host genome integrity. Cell Host Microbe.

[110] Ahmad R., Kumar B., Chen Z., Chen X., Muller D., Lele S.M., Washington M.K., Batra S.K., Dhawan P., Singh A.B. (2017). Loss of claudin-3 expression induces IL6/gp130/Stat3 signaling to promote colon cancer malignancy by hyperactivating Wnt/β-catenin signaling. Oncogene.

[111] Schirmer M., Smeekens S.P., Vlamakis H., Jaeger M., Oosting M., Franzosa E.A., Ter Horst R., Jansen T., Jacobs L., Bonder M.J. (2016). Linking the Human Gut Microbiome to Inflammatory Cytokine Production Capacity. Cell.

[112] Mousa S., Sarfraz M., Mousa W.K. (2023). The Interplay between Gut Microbiota and Oral Medications and Its Impact on Advancing Precision Medicine. Metabolites.

[113] Dziubanska-Kusibab P.J., Berger H., Battistini F., Bouwman B.A.M., Iftekhar A., Katainen R., Cajuso T., Crosetto N., Orozco M., Aaltonen L.A. (2020). Colibactin DNA-damage signature indicates mutational impact in colorectal cancer. Nat. Med..

[114] Xu F., Li Q., Wang S., Dong M., Xiao G., Bai J., Wang J., Sun X. (2023). The efficacy of prevention for colon cancer based on the microbiota therapy and the antitumor mechanisms with intervention of dietary Lactobacillus. Microbiol. Spectr..

[115] Garajova I., Balsano R., Wang H., Leonardi F., Giovannetti E., Deng D., Peters G.J. (2021). The role of the microbiome in drug resistance in gastrointestinal cancers. Expert Rev. Anticancer. Ther..

[116] Ocvirk S., O’Keefe S.J. (2017). Influence of Bile Acids on Colorectal Cancer Risk: Potential Mechanisms Mediated by Diet—Gut Microbiota Interactions. Curr. Nutr. Rep..

[117] Jin D., Huang K., Xu M., Hua H., Ye F., Yan J., Zhang G., Wang Y. (2022). Deoxycholic acid induces gastric intestinal metaplasia by activating STAT3 signaling and disturbing gastric bile acids metabolism and microbiota. Gut Microbes.

[118] Khalaf K., Hana D., Chou J.T., Singh C., Mackiewicz A., Kaczmarek M. (2021). Aspects of the Tumor Microenvironment Involved in Immune Resistance and Drug Resistance. Front. Immunol..

[119] Geller L.T., Barzily-Rokni M., Danino T., Jonas O.H., Shental N., Nejman D., Gavert N., Zwang Y., Cooper Z.A., Shee K. (2017). Potential role of intratumor bacteria in mediating tumor resistance to the chemotherapeutic drug gemcitabine. Science.

[120] Alexander J.L., Wilson I.D., Teare J., Marchesi J.R., Nicholson J.K., Kinross J.M. (2017). Gut microbiota modulation of chemotherapy efficacy and toxicity. Nat. Rev. Gastroenterol. Hepatol..

[121] Colbert L.E., El Alam M.B., Wang R., Karpinets T., Lo D., Lynn E.J., Harris T.A., Elnaggar J.H., Yoshida-Court K., Tomasic K. (2023). Tumor-resident Lactobacillus iners confer chemoradiation resistance through lactate-induced metabolic rewiring. Cancer Cell.

[122] Aykut B., Pushalkar S., Chen R., Li Q., Abengozar R., Kim J.I., Shadaloey S.A., Wu D., Preiss P., Verma N. (2019). The fungal mycobiome promotes pancreatic oncogenesis via activation of MBL. Nature.

[123] Mohindroo C., Hasanov M., Rogers J.E., Dong W., Prakash L.R., Baydogan S., Mizrahi J.D., Overman M.J., Varadhachary G.R., Wolff R.A. (2021). Antibiotic use influences outcomes in advanced pancreatic adenocarcinoma patients. Cancer Med..

[124] Nakano S., Komatsu Y., Kawamoto Y., Saito R., Ito K., Nakatsumi H., Yuki S., Sakamoto N. (2020). Association between the use of antibiotics and efficacy of gemcitabine plus nab-paclitaxel in advanced pancreatic cancer. Medicine.

[125] Sivan A., Corrales L., Hubert N., Williams J.B., Aquino-Michaels K., Earley Z.M., Benyamin F.W., Lei Y.M., Jabri B., Alegre M.L. (2015). Commensal Bifidobacterium promotes antitumor immunity and facilitates anti-PD-L1 efficacy. Science.

[126] Lee C.G., Hwang S., Gwon S.Y., Park C., Jo M., Hong J.E., Rhee K.J. (2022). Bacteroides fragilis Toxin Induces Intestinal Epithelial Cell Secretion of Interleukin-8 by the E-Cadherin/β-Catenin/NF-κB Dependent Pathway. Biomedicines.

[127] Geis A.L., Fan H., Wu X., Wu S., Huso D.L., Wolfe J.L., Sears C.L., Pardoll D.M., Housseau F. (2015). Regulatory T-cell Response to Enterotoxigenic Bacteroides fragilis Colonization Triggers IL17-Dependent Colon Carcinogenesis. Cancer Discov..

[128] Bender M.J., McPherson A.C., Phelps C.M., Pandey S.P., Laughlin C.R., Shapira J.H., Medina Sanchez L., Rana M., Richie T.G., Mims T.S. (2023). Dietary tryptophan metabolite released by intratumoral Lactobacillus reuteri facilitates immune checkpoint inhibitor treatment. Cell.

[129] Tan J. (2015). Immunotherapy Meets Microbiota. Cell.

[130] Goc J., Sonnenberg G.F. (2022). Harnessing Microbiota to Improve Immunotherapy for Gastrointestinal Cancers. Cancer Immunol. Res..

[131] Routy B., Gopalakrishnan V., Daillere R., Zitvogel L., Wargo J.A., Kroemer G. (2018). The gut microbiota influences anticancer immunosurveillance and general health. Nat. Rev. Clin. Oncol..

[132] Utz V.E.M., Perdigon G., LeBlanc A.d.M.d. (2019). Milk fermented by Lactobacillus casei CRL431 modifies cytokine profiles associated to different stages of breast cancer development in mice. Benef. Microbes.

[133] Kang X., Liu C., Ding Y., Ni Y., Ji F., Lau H.C.H., Jiang L., Sung J.J., Wong S.H., Yu J. (2023). Roseburia intestinalis generated butyrate boosts anti-PD-1 efficacy in colorectal cancer by activating cytotoxic CD8(+) T cells. Gut.

[134] Ramaiah M.J., Tangutur A.D., Manyam R.R. (2021). Epigenetic modulation and understanding of HDAC inhibitors in cancer therapy. Life Sci..

[135] Li J., Zhang Y., Cai Y., Yao P., Jia Y., Wei X., Du C., Zhang S. (2024). Multi-omics analysis elucidates the relationship between intratumor microbiome and host immune heterogeneity in breast cancer. Microbiol. Spectr..

[136] Jin M., Shang F., Wu J., Fan Q., Chen C., Fan J., Liu L., Nie X., Zhang T., Cai K. (2021). Tumor-Associated Microbiota in Proximal and Distal Colorectal Cancer and Their Relationships with Clinical Outcomes. Front. Microbiol..

[137] Banerjee S., Wei Z., Tian T., Bose D., Shih N.N.C., Feldman M.D., Khoury T., De Michele A., Robertson E.S. (2021). Prognostic correlations with the microbiome of breast cancer subtypes. Cell Death Dis..

[138] Newsome R.C., Jobin C. (2021). Microbiome-Derived Liquid Biopsy: New Hope for Cancer Screening?. Clin. Chem..

[139] Leng Q., Holden V.K., Deepak J., Todd N.W., Jiang F. (2021). Microbiota Biomarkers for Lung Cancer. Diagnostics.

[140] Yuan X., Wang Z., Li C., Lv K., Tian G., Tang M., Ji L., Yang J. (2022). Bacterial biomarkers capable of identifying recurrence or metastasis carry disease severity information for lung cancer. Front. Microbiol..

[141] Zhang C., Wang J., Sun Z., Cao Y., Mu Z., Ji X. (2021). Commensal microbiota contributes to predicting the response to immune checkpoint inhibitors in non-small-cell lung cancer patients. Cancer Sci..

[142] Qin H., Liu J., Qu Y., Li Y.Y., Xu Y.L., Yan Y.F. (2024). The intratumoral microbiota biomarkers for predicting survival and efficacy of immunotherapy in patients with ovarian serous cystadenocarcinoma. J. Ovarian Res..

[143] Liu N.N., Yi C.X., Wei L.Q., Zhou J.A., Jiang T., Hu C.C., Wang L., Wang Y.Y., Zou Y., Zhao Y.K. (2023). The intratumor mycobiome promotes lung cancer progression via myeloid-derived suppressor cells. Cancer Cell.

[144] Boesch M., Baty F., Albrich W.C., Flatz L., Rodriguez R., Rothschild S.I., Joerger M., Fruh M., Brutsche M.H. (2021). Local tumor microbial signatures and response to checkpoint blockade in non-small cell lung cancer. Oncoimmunology.

[145] Zhao L., Grimes S.M., Greer S.U., Kubit M., Lee H., Nadauld L.D., Ji H.P. (2021). Characterization of the consensus mucosal microbiome of colorectal cancer. NAR Cancer.

[146] Eckhoff A.M., Fletcher A.A., Kelly M.S., Dohlman A., McIntyre C.A., Shen X., Iyer M.K., Nussbaum D.P., Allen P.J. (2024). Comprehensive Assessment of the Intrinsic Pancreatic Microbiome. Ann. Surg..

[147] Dong J., Gao H.L., Wang W.Q., Yu X.J., Liu L. (2021). Bidirectional and dynamic interaction between the microbiota and therapeutic resistance in pancreatic cancer. Biochim. Biophys. Acta Rev. Cancer.

[148] Wu T., Dai Y. (2017). Tumor microenvironment and therapeutic response. Cancer Lett..

[149] Rescigno M. (2023). Training the microbiota to increase immune checkpoint blockade and to reduce toxicity. Eur. J. Immunol..

[150] Ducarmon Q.R., Hornung B.V.H., Geelen A.R., Kuijper E.J., Zwittink R.D. (2020). Toward Standards in Clinical Microbiota Studies: Comparison of Three DNA Extraction Methods and Two Bioinformatic Pipelines. mSystems.

[151] Bharti R., Grimm D.G. (2021). Current challenges and best-practice protocols for microbiome analysis. Brief. Bioinform..

[152] Hennessy M., Morais E.S., Devoy C., Walsh G., Crowley E., Lynch E., Twomey M., Girleanu C., Dahly D.L., O’Driscoll L. (2024). Prospective evaluation of the breast microbiome and tumor microenviron ment-related biomarkers of response to neoadjuvant systemic therapy in triple negative breast cancer. J. Clin. Oncol..

[153] Mandrekar S.J., Sargent D.J. (2010). Predictive biomarker validation in practice: Lessons from real trials. Clin. Trials.

[154] Owokotomo O.E., Sengupta R., Shkedy Z. (2023). Development of Microbiome Biomarkers in Intervention Studies. J. Appl. Microbiol..

[155] Song W., Anselmo A.C., Huang L. (2019). Nanotechnology intervention of the microbiome for cancer therapy. Nat. Nanotechnol..

[156] Danino T., Prindle A., Kwong G.A., Skalak M., Li H., Allen K., Hasty J., Bhatia S.N. (2015). Programmable probiotics for detection of cancer in urine. Sci. Transl. Med..

[157] Gurbatri C.R., Arpaia N., Danino T. (2022). Engineering bacteria as interactive cancer therapies. Science.

[158] Chen F., Zang Z., Chen Z., Cui L., Chang Z., Ma A., Yin T., Liang R., Han Y., Wu Z. (2019). Nanophotosensitizer-engineered Salmonella bacteria with hypoxia targeting and photothermal-assisted mutual bioaccumulation for solid tumor therapy. Biomaterials.

[159] Geng Z., Cao Z., Liu R., Liu K., Liu J., Tan W. (2021). Aptamer-assisted tumor localization of bacteria for enhanced biotherapy. Nat. Commun..

[160] Nguyen V.H., Kim H.S., Ha J.M., Hong Y., Choy H.E., Min J.J. (2010). Genetically engineered Salmonella typhimurium as an imageable therapeutic probe for cancer. Cancer Res..

[161] Cronin M., Akin A.R., Collins S.A., Meganck J., Kim J.B., Baban C.K., Joyce S.A., van Dam G.M., Zhang N., van Sinderen D. (2012). High resolution in vivo bioluminescent imaging for the study of bacterial tumour targeting. PLoS ONE.

[162] Jiang S.N., Park S.H., Lee H.J., Zheng J.H., Kim H.S., Bom H.S., Hong Y., Szardenings M., Shin M.G., Kim S.C. (2013). Engineering of bacteria for the visualization of targeted delivery of a cytolytic anticancer agent. Mol. Ther..

[163] Bourdeau R.W., Lee-Gosselin A., Lakshmanan A., Farhadi A., Kumar S.R., Nety S.P., Shapiro M.G. (2018). Acoustic reporter genes for noninvasive imaging of microorganisms in mammalian hosts. Nature.

[164] Din M.O., Danino T., Prindle A., Skalak M., Selimkhanov J., Allen K., Julio E., Atolia E., Tsimring L.S., Bhatia S.N. (2016). Synchronized cycles of bacterial lysis for in vivo delivery. Nature.

[165] Chowdhury S., Castro S., Coker C., Hinchliffe T.E., Arpaia N., Danino T. (2019). Programmable bacteria induce durable tumor regression and systemic antitumor immunity. Nat. Med..

[166] Gurbatri C.R., Lia I., Vincent R., Coker C., Castro S., Treuting P.M., Hinchliffe T.E., Arpaia N., Danino T. (2020). Engineered probiotics for local tumor delivery of checkpoint blockade nanobodies. Sci. Transl. Med..

[167] Gujrati V., Kim S., Kim S.H., Min J.J., Choy H.E., Kim S.C., Jon S. (2014). Bioengineered bacterial outer membrane vesicles as cell-specific drug-delivery vehicles for cancer therapy. ACS Nano.

[168] Hasan S., Thomas N., Thierry B., Prestidge C.A. (2017). Controlled and Localized Nitric Oxide Precursor Delivery from Chitosan Gels to Staphylococcus aureus Biofilms. J. Pharm. Sci..

[169] Montanaro J., Inic-Kanada A., Ladurner A., Stein E., Belij S., Bintner N., Schlacher S., Schuerer N., Mayr U.B., Lubitz W. (2015). Escherichia coli Nissle 1917 bacterial ghosts retain crucial surface properties and express chlamydial antigen: An imaging study of a delivery system for the ocular surface. Drug Des. Dev. Ther..

[170] Kim O.Y., Dinh N.T., Park H.T., Choi S.J., Hong K., Gho Y.S. (2017). Bacterial protoplast-derived nanovesicles for tumor targeted delivery of chemotherapeutics. Biomaterials.

[171] Qing S., Lyu C., Zhu L., Pan C., Wang S., Li F., Wang J., Yue H., Gao X., Jia R. (2020). Biomineralized Bacterial Outer Membrane Vesicles Potentiate Safe and Efficient Tumor Microenvironment Reprogramming for Anticancer Therapy. Adv. Mater..

[172] Jiang Y., Zhou Z., Liu C., Wang L., Li C. (2023). Bacterial outer membrane vesicles as drug delivery carrier for photodynamic anticancer therapy. Front. Chem..

[173] Kuerban K., Gao X., Zhang H., Liu J., Dong M., Wu L., Ye R., Feng M., Ye L. (2020). Doxorubicin-loaded bacterial outer-membrane vesicles exert enhanced anti-tumor efficacy in non-small-cell lung cancer. Acta Pharm. Sin. B.

[174] Huang Y., Beringhs A.O., Chen Q., Song D., Chen W., Lu X., Fan T.H., Nieh M.P., Lei Y. (2019). Genetically Engineered Bacterial Outer Membrane Vesicles with Expressed Nanoluciferase Reporter for in Vivo Bioluminescence Kinetic Modeling through Noninvasive Imaging. ACS Appl. Bio Mater..

[175] Li Y., Cong Z., Xie L., Tang S., Ren C., Peng X., Tang D., Wan F., Han H., Zhang X. (2023). Magnetically Powered Immunogenic Macrophage Microrobots for Targeted Multimodal Cancer Therapy. Small.

[176] Szollosi D., Hajdrik P., Tordai H., Bergmann R., Horvath I., Mihaly J., Gaal A., Jezso B., Shailaja K.D., Felfoldi T. (2024). Quantitative Biodistribution of OMVs Using SPECT/CT Imaging with HYNIC-Duramycin Radiolabeling. ACS Omega.

[177] Li Y., Zhao R., Cheng K., Zhang K., Wang Y., Zhang Y., Li Y., Liu G., Xu J., Xu J. (2020). Bacterial Outer Membrane Vesicles Presenting Programmed Death 1 for Improved Cancer Immunotherapy via Immune Activation and Checkpoint Inhibition. ACS Nano.

[178] Sun J., Tan L., Ye B.C., Bi X. (2024). Engineered Outer Membrane Vesicles as Nanosized Immune Cell Engagers for Enhanced Solid Tumor Immunotherapy. ACS Nano.

[179] Kieser S., Zdobnov E.M., Trajkovski M. (2022). Comprehensive mouse microbiota genome catalog reveals major difference to its human counterpart. PLoS Comput. Biol..

[180] Krych L., Hansen C.H., Hansen A.K., van den Berg F.W., Nielsen D.S. (2013). Quantitatively different, yet qualitatively alike: A meta-analysis of the mouse core gut microbiome with a view towards the human gut microbiome. PLoS ONE.

[181] Daillere R., Vetizou M., Waldschmitt N., Yamazaki T., Isnard C., Poirier-Colame V., Duong C.P.M., Flament C., Lepage P., Roberti M.P. (2016). Enterococcus hirae and Barnesiella intestinihominis Facilitate Cyclophosphamide-Induced Therapeutic Immunomodulatory Effects. Immunity.

[182] Fluckiger A., Daillere R., Sassi M., Sixt B.S., Liu P., Loos F., Richard C., Rabu C., Alou M.T., Goubet A.G. (2020). Cross-reactivity between tumor MHC class I-restricted antigens and an enterococcal bacteriophage. Science.

[183] Thomas R.M., Jobin C. (2015). The Microbiome and Cancer: Is the ’Oncobiome’ Mirage Real?. Trends Cancer.

[184] Le D.T., Wang-Gillam A., Picozzi V., Greten T.F., Crocenzi T., Springett G., Morse M., Zeh H., Cohen D., Fine R.L. (2015). Safety and survival with GVAX pancreas prime and Listeria Monocytogenes-expressing mesothelin (CRS-207) boost vaccines for metastatic pancreatic cancer. J. Clin. Oncol..

[185] Basu P., Mehta A., Jain M., Gupta S., Nagarkar R.V., John S., Petit R. (2018). A Randomized Phase 2 Study of ADXS11-001 Listeria monocytogenes-Listeriolysin O Immunotherapy with or Without Cisplatin in Treatment of Advanced Cervical Cancer. Int. J. Gynecol. Cancer.

[186] Sawant S.S., Patil S.M., Gupta V., Kunda N.K. (2020). Microbes as Medicines: Harnessing the Power of Bacteria in Advancing Cancer Treatment. Int. J. Mol. Sci..

[187] Zheng J.H., Min J.J. (2016). Targeted Cancer Therapy Using Engineered Salmonella typhimurium. Chonnam Med. J..

[188] Nallar S.C., Xu D.Q., Kalvakolanu D.V. (2017). Bacteria and genetically modified bacteria as cancer therapeutics: Current advances and challenges. Cytokine.

[189] Ma Y., Chen H., Li H., Zheng M., Zuo X., Wang W., Wang S., Lu Y., Wang J., Li Y. (2024). Intratumor microbiome-derived butyrate promotes lung cancer metastasis. Cell Rep. Med..

[190] Pauli C., Hopkins B.D., Prandi D., Shaw R., Fedrizzi T., Sboner A., Sailer V., Augello M., Puca L., Rosati R. (2017). Personalized In Vitro and In Vivo Cancer Models to Guide Precision Medicine. Cancer Discov..

[191] Zanella E.R., Grassi E., Trusolino L. (2022). Towards precision oncology with patient-derived xenografts. Nat. Rev. Clin. Oncol..

[192] Chupp D.P., Rivera C.E., Zhou Y., Xu Y., Ramsey P.S., Xu Z., Zan H., Casali P. (2024). A humanized mouse that mounts mature class-switched, hypermutated and neutralizing antibody responses. Nat. Immunol..

[193] Sontheimer-Phelps A., Hassell B.A., Ingber D.E. (2019). Modelling cancer in microfluidic human organs-on-chips. Nat. Rev. Cancer.

[194] Ballerini M., Galie S., Tyagi P., Catozzi C., Raji H., Nabinejad A., Macandog A.D.G., Cordiale A., Slivinschi B.I., Kugiejko K.K. (2025). A gut-on-a-chip incorporating human faecal samples and peristalsis predicts responses to immune checkpoint inhibitors for melanoma. Nat. Biomed. Eng..

